# Photocatalytic H_2_O_2_ Production over Ultrathin Layered Double Hydroxide with 3.92% Solar-to-H_2_O_2_ Efficiency

**DOI:** 10.1007/s40820-025-02044-0

**Published:** 2026-01-12

**Authors:** Yamin Xi, Zechun Lu, Tong Bao, Yingying Zou, Chaoqi Zhang, Chunhong Xia, Guangfeng Wei, Chengzhong Yu, Chao Liu

**Affiliations:** 1 School of Chemistry and Molecular Engineering, East China Normal University, Shanghai, 200241 People’s Republic of China; 2 State Key Laboratory of Petroleum Molecular and Process Engineering, SKLPMPE, East China Normal University, Shanghai, 200241 People’s Republic of China; 3 Shanghai Key Laboratory of Chemical Assessment and Sustainability, School of Chemical, Science and Engineering, Tongji University, Shanghai, 200092 People’s Republic of China; 4 Australian Institute for Bioengineering and Nanotechnology, The University of Queensland, Brisbane, QLD 4072 Australia; 5 Shanghai Frontiers Science Center of Molecule Intelligent Syntheses, School of Chemistry and Molecular Engineering, East China Normal University, Shanghai, 200241 People’s Republic of China

**Keywords:** Layered double hydroxide, Intercalated nitrate, Facet, Photocatalysis, Hydrogen peroxide

## Abstract

Artificial photosynthesis of hydrogen peroxide (H_2_O_2_) from earth-abundant water and oxygen is a sustainable approach, however current photocatalysts suffer from low production rate and solar-to-chemical conversion efficiency (< 1.5%). Herein, we report that nickel–chromium layered double hydroxide with intercalated nitrate (NiCrOOH-NO_3_) and a thickness of ~ 4.4 nm is an efficient photocatalyst, enabling a H_2_O_2_ production yield of 28.7 mmol g^−1^ h^−1^ under visible light irradiation with 3.92% solar-to-chemical conversion efficiency. Experimental and computational studies have revealed an inherent facet-dependent reduction–oxidation reaction behavior and spatial separation of photogenerated electrons and holes. An unexpected role of intercalated nitrate is demonstrated, which promotes excited electron—hole spatial separation and facilitates the electron transfer to oxygen intermediate via delocalization. This work provides understandings in the impact of nanostructure and anion in the design of advanced photocatalysts, paving the way toward practical synthesis of H_2_O_2_ using fully solar-driven renewable energy.

**Supplementary Information:**

The online version contains supplementary material available at 10.1007/s40820-025-02044-0.

## Highlights

- The use of layered double hydroxides for photocatalytic for H_2_O_2_ production is innovatively demonstrated.
- Facet-dependent spatial charge separation enables maximized carrier utilization efficiency.
- The unique role of intercalated nitrate in promoting electron-hole separation and facilitating intermolecular electron transfer is unveiled.
- A record-high H_2_O_2_ production rate of 28.7 mmol g^-1^ h^-1^ with 3.92% solar-to-chemical efficiency is achieved.

**Supplementary Information:**

The online version contains supplementary material available at 10.1007/s40820-025-02044-0.

## Graphical abstract

**Figure:**
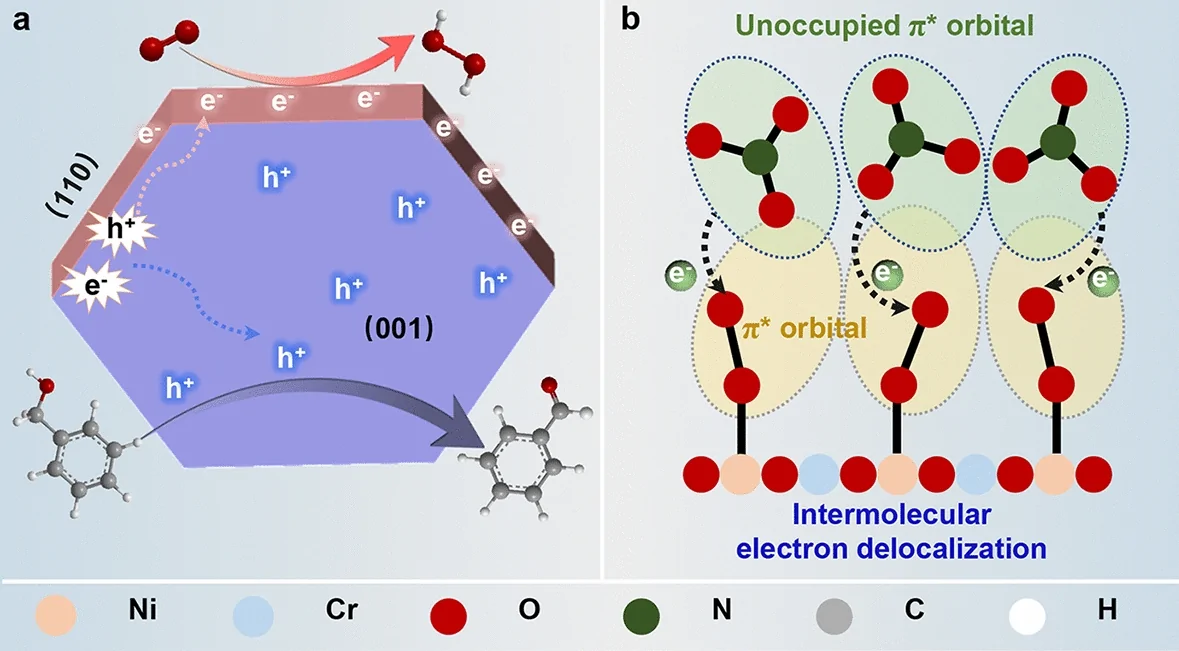

## Introduction

Hydrogen peroxide (H_2_O_2_) is an essential chemical with broad applications in chemical synthesis, environmental protection, medical disinfection, etc. [1, 2]. The industrial production of H_2_O_2_ is predominately achieved by the anthraquinone process, which suffers from high energy consumption and waste discharge [3]. As an alternative, the photocatalytic two-electron oxygen reduction reaction (2e^−^ ORR) driven by solar energy emerges as a direct, contaminant-free and sustainable approach for on-site H_2_O_2_ production [4]. To date, numerous semiconducting materials such as metal sulfides [5], metal oxides [6], graphitic carbon nitride (g-C_3_N_4_) [7], metal–organic frameworks (MOFs) [8] and covalent organic frameworks (COFs) [9] have been reported for H_2_O_2_ production. However, the overall performance is unsatisfactory with low H_2_O_2_ production rate (< 20 mmol g^−1^ h^−1^) and low solar-to-chemical conversion (SCC) efficiency (< 2.3% with only solar energy input), mainly due to insufficient utilization of photogenerated carriers and charge separation [10]. The development of effective 2e^−^ ORR photocatalysts for H_2_O_2_ production remains a challenge.

Ultrathin two-dimensional (2D) nanomaterials constitute an important class of advanced nanomaterials, featuring sheet-like structures with the lateral size over 100 nm but the thickness typically less than 5 nm [11, 12]. A plenty of ultrathin 2D nanomaterials such as graphitic carbon nitride [13], transition metal dichalcogenides [14], bismuth oxybromide [15], covalent-organic frameworks [16], and metal phosphorus trichalcogenides [17] have been fabricated with extraordinary properties and versatile applications. Layered double hydroxides (LDHs) are another type of 2D nanomaterials and photocatalysts [18]. Known as hydrotalcite-like anionic clay, LDHs are comprised of divalent and trivalent metal cations coordinated with hydroxide ions and intercalated with charge-compensating anions (e.g., NO_3_^−^, Cl^−^) [19, 20]. To date, LDHs have been extensively studied as photocatalysts for water splitting [20], CO_2_ reduction [21], N_2_ fixation [22] and pollutant degradation [23], however their use in photocatalytic H_2_O_2_ production is rarely reported. Moreover, in the pursuit of high-performance LDH-based photocatalysts with various structural modification strategies (e.g., heterometal doping [24], defect engineering [25], cocatalyst loading [26] and heterojunction construction [27]), the impact of the intrinsic nanostructure (e.g., well-defined facet exposure) and interlayered anion on the photocatalytic properties of LDHs is largely overlooked.

**Scheme 1:**
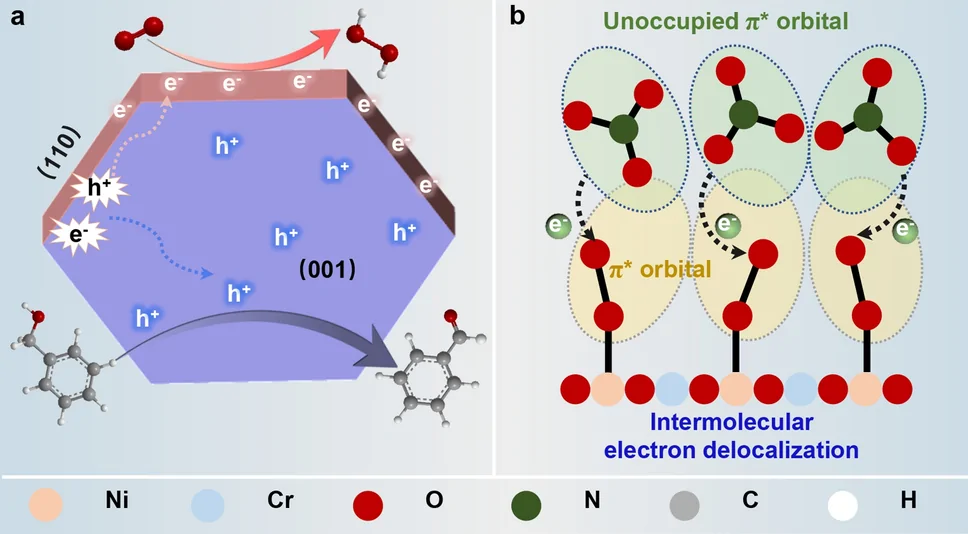
Schematic illustration of **a** facet-dependent redox reaction behavior and **b** intermolecular electron delocalization between NO_3_^−^ and adsorbed O_2_ of NiCrOOH-NO_3_

Herein, we report nitrate anion (NO_3_^−^) intercalated NiCr-LDH ultrathin nanosheets (NiCrOOH-NO_3_) as a highly efficient photocatalyst for 2e^−^ ORR to H_2_O_2_ coupled with benzyl alcohol oxidation reaction (BAOR) to benzaldehyde (BAD). Experimental results combined with theoretical calculations reveal the facet-dependent catalytic behavior with 2e^−^ ORR on (110) planes and BAOR on (001) planes, inducing the directional migration of photogenerated carriers and enhancing the charge separation (Scheme 1a). Besides, the intermolecular electron delocalization between unoccupied π* orbital of interlayered NO_3_^−^ and π* orbital of adsorbed O_2_ can promote the formation of electron–hole pairs and facilitate the transfer of photogenerated electron to oxygen intermediate (Scheme 1b). By virtue of the rational design at both nano and molecular scales, the NiCrOOH-NO_3_ photocatalyst exhibits a record-high performance with a H_2_O_2_ production rate of 28.7 mmol g^−1^ h^−1^ and a SCC efficiency of 3.92% with only solar energy input. For the coupled BAOR, a high BAD yield of 27.6 mmol g^−1^ h^−1^ is achieved. Notably, both the 2e^−^ ORR and BAOR performances are superior than the reported photocatalysts.

## Experimental Section

### Materials

Ni(NO_3_)_2_·6H_2_O, Cr(NO_3_)_3_·9H_2_O, NiCl_2_·6H_2_O, CrCl_3_·6H_2_O and C_8_H_5_KO_4_ were obtained from Shanghai Titan Scientific Co., Ltd. Benzyl alcohol (BA, AR), KI and NaOH were purchased from Sigma-Aldrich. 30% hydrogen peroxide aqueous solution was supplied by Sinopharm Chemical Reagent Co., Ltd. All chemicals were used as received without further purification. Doubly distilled water was used in all experiments.

### Preparation of NiCrOOH-NO_3_, NiCrOOH-NO_3_-T and NiCrOOH-Cl Nanosheets

#### Synthesis of NiCrOOH-NO_3_ Nanosheets

5 mmol of Ni(NO_3_)_2_·6H_2_O and 2.5 mmol of Cr(NO_3_)_3_·9H_2_O were dissolved in 9 mL of deionized water (solution A). NaOH was dissolved in 9 mL of deionized water to give a 3 M alkaline solution (solution B). Then, solution B was rapidly added into solution A and stirred rapidly at room temperature for 1 min. Then, the mixed solution was transferred into a Teflon-lined stainless-steek autoclave and kept at 120 °C for 12 h. After cooling down to room temperature, the solid products were collected by centrifugation, washed three times with deionized water and ethanol, and drying overnight. The final products were obtained and denoted as NiCrOOH-NO_3_.

#### Synthesis of NiCrOOH-NO_3_-T Nanosheets

NiCrOOH-NO_3_-T was prepared by the similar procedure except that the reaction time was extended to 48 h. Through the same washing process of NiCrOOH-NO_3_, the sample NiCrOOH-NO_3_-T was obtained.

#### Synthesis of NiCrOOH-Cl Nanosheets

NiCrOOH-Cl was prepared by the similar procedure except that the NiCl_2_·6H_2_O, CrCl_3_·6H_2_O were used as the starting materials. After reaction for 12 h at 120 °C, the NiCrOOH-Cl products were collected, washed with deionized water and ethanol for four times and dried overnight.

## Results and Discussion

### Structural Characterizations

**Fig. 1:**
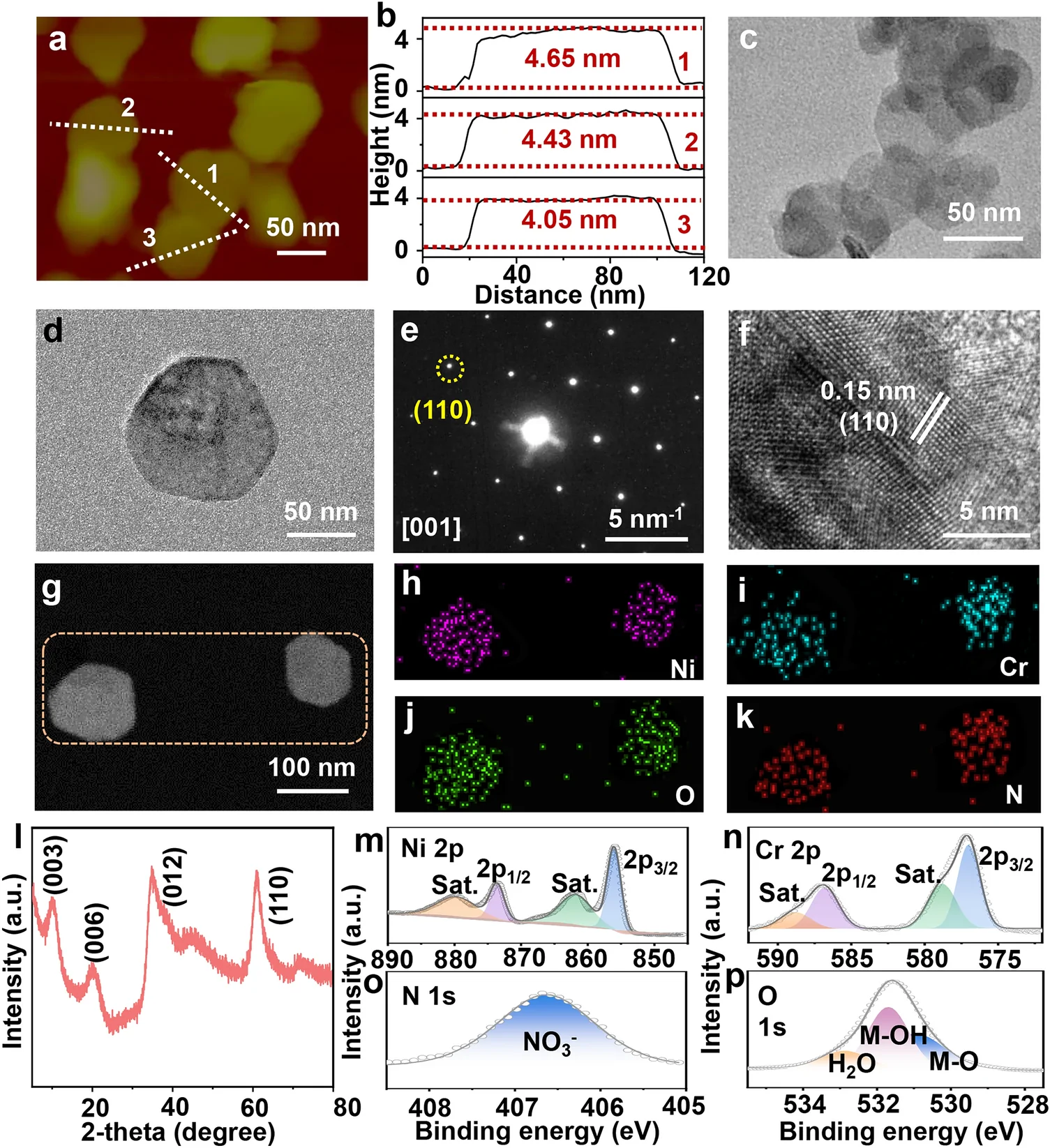
**a** AFM image and **b** height profiles of NiCrOOH-NO_3_ nanosheets. **c**, **d** TEM, **e**, **f** SAED and HRTEM, **g** HADDF-STEM and element mapping images, **l** XRD pattern of NiCrOOH-NO_3_ nanosheets. High-resolution XPS spectra of **m** Ni 2*p*, **n** Cr 2*p*,** o** N 1*s* and **p** O 1*s* of NiCrOOH-NO_3_

Ultrathin NiCrOOH-NO_3_ nanosheets were prepared using mixed metal nitrate precursors and NaOH as a co-precipitation agent. Atomic force microscopy (AFM) and transmission electron microscopy (TEM) techniques were used to characterize the morphology and structure of NiCrOOH-NO_3_. AFM image shows that NiCrOOH-NO_3_ possesses a uniform nanosheet morphology (Fig. 1a), the height profiles reveal an average thickness of ~ 4.4 nm (Fig. 1b). Under TEM observation, NiCrOOH-NO_3_ displays a flake-like structure with an average diameter of ~ 100 nm (Fig. 1c) and a nearly hexagonal shape (Fig. 1d). The selected-area electron diffraction (SAED) pattern reveals hexagonally arrayed spots, indicating a highly ordered hexagonal structure along the [001] direction (Fig. 1e). By corelating the TEM image and SAED pattern, the side edges of the nanosheet are identified as the {110} planes of NiCrOOH-NO_3_. In the high-resolution TEM (HRTEM) image (Fig. 1f), the clear lattice fringes with planar distance of 0.15 nm are also assigned to the (110) planes of NiCrOOH-NO_3_ [21]. Further, under scanning transmission electron microscopy (STEM) mode, the high-angle annular dark-field (HAADF) and corresponding energy-dispersive X-ray spectroscopy (EDX) elemental mapping images demonstrate the homogeneous distribution of Ni, Cr, N, and O elements in the NiCrOOH-NO_3_ nanosheets with a hexagonal shape (Fig. 1g–k).

The crystallinity of NiCrOOH-NO_3_ is characterized using X-ray diffraction (XRD). As shown in the XRD pattern (Fig. 1l), the peaks of NiCrOOH-NO_3_ observed at 2θ = 10.18°, 21.3°, 34.7°, and 60.6° can be indexed to the (003), (006), (012), and (110) diffractions of a hydrotalcite-like structure (PDF#52–1626) [28]. The interlayer spacing (d_003_) of NiCrOOH-NO_3_ is calculated to be 0.87 nm, indicating that one NiCrOOH-NO_3_ nanosheet is consisted of five layers. The chemical composition and valence state of NiCrOOH-NO_3_ were analyzed by X-ray photoelectron spectroscopy (XPS). In the XPS survey spectrum of NiCrOOH-NO_3_ (Fig. S1, Table S1), Ni, Cr, O, and N elements were detected with a NO_3_^−^/metal molar ratio of ~ 0.33. In the Ni 2*p* spectrum of NiCrOOH-NO_3_ (Fig. 1m), two peaks of 2*p*_3/2_ and 2*p*_1/2_ states of Ni^2+^ at 856.12 and 873.76 eV are deconvoluted, with two satellite peaks at 861.76 and 879.72 eV. The Cr 2*p* spectrum (Fig. 1n) exhibits two peaks assigned to 2*p*_3/2_ and 2*p*_1/2_ orbitals of Cr^3+^ at 577.00 and 586.87 eV, and two satellite peaks at 578.82 and 588.78 eV, respectively. For N 1*s* spectrum (Fig. 1o), the peak located at 406.55 eV is attributed to the intercalated NO_3_^−^ ions. Figure 1p displays the O 2*p* spectrum, where two peaks corresponding to the lattice H_2_O, metal-hydroxyl (M–OH) and M–O bond can be deconvoluted at 532.87, 531.69 and 530.95 eV. The attenuated total reflectance Fourier transform infrared (ATR-FTIR) spectrum of NiCrOOH-NO_3_ is presented in Fig. S2. The broad band located at 3496 cm^−1^ belongs to the O–H stretching vibration from interlayer water molecules in LDH [29]. Besides, the sharp bands at 1634 and 1335 cm^−1^ are attributed to the vibration modes of hydroxyl groups and interlayer NO_3_^−^ anions, respectively.

Thermogravimetric analysis (TGA) was performed to further study the chemical compositions. In the TGA curve of NiCrOOH-NO_3_ (Fig. S3), the first-stage weight loss of ≈ 12.97% at 100–180 °C is associated with the removal of interlayer lattice H_2_O. The shrinkage of hydroxide layers results in the second-stage weight loss of ≈ 14.10% between 180 and 280 °C. The further weight loss of 12.00% after 280 °C is ascribed to the decomposition of NO_3_^−^ anions [28, 30]. According to TGA analyses, the formulas of NiCrOOH-NO_3_ can be calculated as Ni_2.00_Cr_0.99_(OH)_5.98_[NO_3_^−^]_0.99_·2.75H_2_O. In addition, the atomic proportion of Ni and Cr is measured to be 2.02 in NiCrOOH-NO_3_ by inductively coupled plasma atomic emission spectrometry (ICP-AES), respectively. In combination with the contents of H, N and O elements determined by elemental analysis (EA, Table S2), the chemical composition of NiCrOOH-NO_3_ was speculated as Ni_2.00_Cr_0.99_N_0.99_O_11.47_H_11.48_, in good agreement with the TGA findings.

For comparison, a thicker nanosheet was synthesized by increasing the reaction time (denoted NiCrOOH-NO_3_-T). As shown in Fig. S4a, b, the average thickness of the NiCrOOH-NO_3_-T is *ca.* 37.9 nm. From the TEM image (Fig. S4c), the hexagonal morphology with a lateral size of ~ 150 nm is clearly seen for NiCrOOH-NO_3_-T. Except for the thickness, the crystalline structure and electronic states of NiCrOOH-NO_3_-T are almost identical with NiCrOOH-NO_3_, as evidenced by the XRD pattern and XPS spectra in Fig. S5. In addition, the Brunauer–Emmett–Teller (BET) specific surface area of NiCrOOH-NO_3_-T was calculated to be 60.2 m^2^ g^−1^ by N_2_ sorption measurements, slightly lower than that of NiCrOOH-NO_3_ (93.1 m^2^ g^−1^, Table S3).

To explore the optical absorption properties and band structures of the samples, ultraviolet–visible diffuse reflectance spectroscopy (UV–Vis DRS) measurement was conducted. As shown in Fig. S7a, the light absorption intensity of NiCrOOH-NO_3_ is stronger than NiCrOOH-NO_3_-T. The absorption band in the region 200–300 nm is attributed to the ligand-to metal charge transfer, *i.e.,* O2*p* → Ni-3*d*-t_2g_/Cr-3*d*-t_2g_. Two more bands located at 420 and 580 nm are assigned to the spin-forbidden transitions (^3^A_2g_(F) → ^1^E_g_(D) and ^3^A_2g_(F) → ^1^T_2g_(D), respectivey) [22, 24]. The bandgaps (E_g_) of NiCrOOH-NO_3_ and NiCrOOH-NO_3_-T were calculated to be 2.37 and 2.48 eV, respectively, using the Kubelka–Munk equation (Fig. S7b). Further according to the Mott–Schottky plots (Fig. S7c), the conduction band (CB) values of NiCrOOH-NO_3_ and NiCrOOH-NO_3_-T are determined as -0.42 and -0.39 V vs. NHE, respectively [31]. Therefore, the valence band (VB) values can be calculated (NiCrOOH-NO_3_: 1.95 eV; NiCrOOH-NO_3_-T: 2.09 eV) and the band structures are illustrated in Fig. S7d. Both samples show sufficient thermodynamic driving force for photocatalytic 2e^−^ ORR and BAOR [32, 33].

### Catalytic Performance Evaluation

**Fig. 2:**
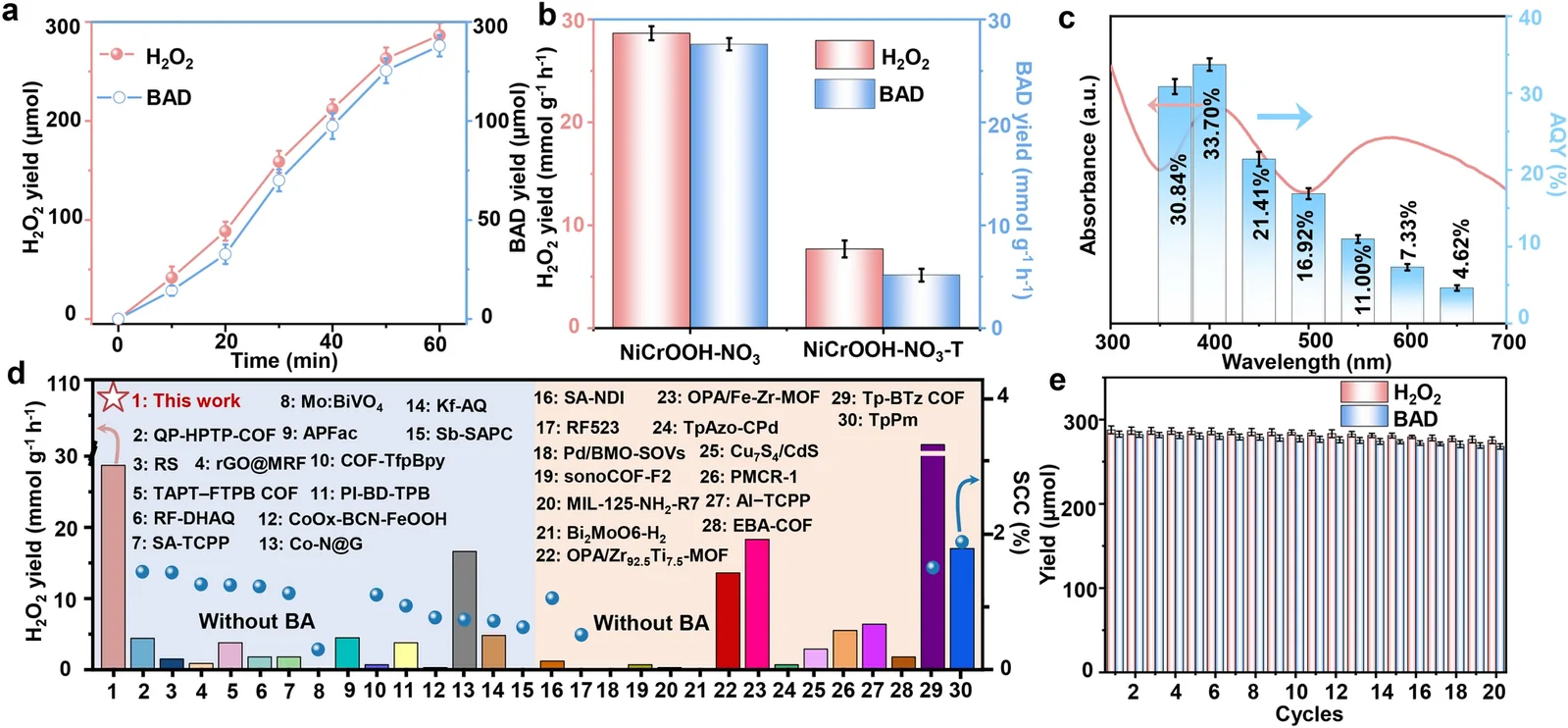
**a** Time course of H_2_O_2_ and BAD contents during the photocatalytic reaction over NiCrOOH-NO_3_. **b** H_2_O_2_ and BAD yield rates of NiCrOOH-NO_3_ and NiCrOOH-NO_3_-T. **c** Wavelength-dependent AQY of NiCrOOH-NO_3_. **d** Comparison of the H_2_O_2_ yield, AQY, and SCC of NiCrOOH-NO_3_ and other recently reported photocatalysts. **e** H_2_O_2_ and BAD yield of NiCrOOH-NO_3_ during recycling stability test

The photocatalytic performance of coupled reaction of 2e^−^ ORR to H_2_O_2_ and BAOR to benzaldehyde (BAD) was evaluated in an oxygen-saturated BA solution under UV–visible light irradiation (320 nm < λ < 780 nm, 0.20 W cm^−2^, Fig. S8). The yields of BAD and H_2_O_2_ were quantified by high-performance liquid chromatography (HPLC) and iodometry method, respectively, and the corresponding standard curves are shown in Fig. S9. The H_2_O_2_ and BAD yields as a function of time are depicted in Figs. 2a and S10. NiCrOOH-NO_3_ produces 286.6 μmol of H_2_O_2_ and 275.9 μmol of BAD in 1 h (Fig. 2a), respectively, significantly higher than those of NiCrOOH-NO_3_-T (76.9 μmol of H_2_O_2_ yield, 51.5 μmol of BAD, Fig. S10), indicating the enhanced activity of NiCrOOH-NO_3_. Furthermore, the H_2_O_2_ yield quantified by colorimetry method is consistent with the result obtained from iodometry method (Figs. S11 and S12). The high H_2_O_2_ production rate of 28.7 mmol g^−1^ h^−1^ (Fig. 2b) is superior to previously reported photocatalysts in both pure water and water-benzyl alcohol systems, and even reaches the level of electrocatalytic 2e^−^ ORR systems (Fig. 2d). For BAD, the yield of 27.6 mmol g^−1^ h^−1^ for NiCrOOH-NO_3_ also exceeds the reported values (Fig. S13). Furthermore, an isotope labeling experiment was conducted by using ^18^O_2_/H_2_^16^O and ^16^O_2_/H₂^18^O as raw materials. Gas chromatography-mass spectrometry (GC–MS) was employed to detect O_2_ generated by the decomposition of H_2_O_2_. In the spectrum recorded by feeding ^18^O_2_ and H_2_^16^O (Fig. S14), a distinct peak assigned to ^18^O_2_ is observed at m/z = 36. When changing the reactants into ^16^O_2_ and H₂^18^O, only a trace amount of ^18^O₂ is detected, indicating that the oxygen in H_2_O_2_ is derived from O_2_.

The light utilization efficiency of NiCrOOH-NO_3_ was also assessed by measuring the apparent quantum yield (AQY). Under the irradiation of monochromatic light from 365 to 650 nm (Fig. 2c), the AQY value of NiCrOOH-NO_3_ follows the same trend with the absorption spectrum. The peak value of AQY reaches 33.7% at 400 nm for NiCrOOH-NO_3_. After correction for scattering and absorption using the integrating sphere method, the absorbed-photon-to-chemical efficiency (APCE) for H₂O₂ production was determined to be 68.78% at 400 nm (Table S4) [34]. Moreover, under the illumination of simulated sunlight (AM 1.5G), the solar conversion chemical efficiency (SCC) of NiCrOOH-NO_3_ was measured to be 3.92% under optimized stirring speed (800 rpm) and optical depth (120 mL of reaction solution) (Figs. S16 and S17). Notably, the SCC efficiency was calculated based on the photocatalytic 2e^−^ ORR pathway specifically for H_2_O_2_ production. Both the AQY and SCC are higher than previously reported photocatalysts (Fig. 2d; Tables S5 and S6) [1, 10, 35–61]. In addition, negligible amounts of H_2_O_2_ were detected in the reaction systems without catalysts or light irradiation, verifying that H_2_O_2_ is predominantly produced from photocatalytic 2e^−^ ORR (Fig. S18). When conducting photocatalysis in Ar-saturated H_2_O, almost no H_2_O_2_ was produced by NiCrOOH-NO_3_ (Fig. S18), excluding the possibility of direct two-electron water oxidation reaction (2e⁻ WOR). Except for high activity, NiCrOOH-NO_3_ also possesses high photocatalytic durability with negligible decline of both ORR and BAOR yields after twenty consecutive reaction cycles with each cycle lasting for 1 h (Fig. 2e). XRD, FTIR and XPS (Figs. S19-S22; Tables S1 and S7) characterizations of NiCrOOH-NO_3_ collected after reaction reveal well-retained morphology, composition and element contents. In the supernatant after reaction, only trace amount of Ni and Cr were detected (Table S7). Furthermore, the nitrate retention in used NiCrOOH-NO_3_ was calculated to be 98.99% according to the XPS results (Table S1), further demonstrating the structural robustness of NiCrOOH-NO_3_.

### Mechanistic Studies

**Fig. 3:**
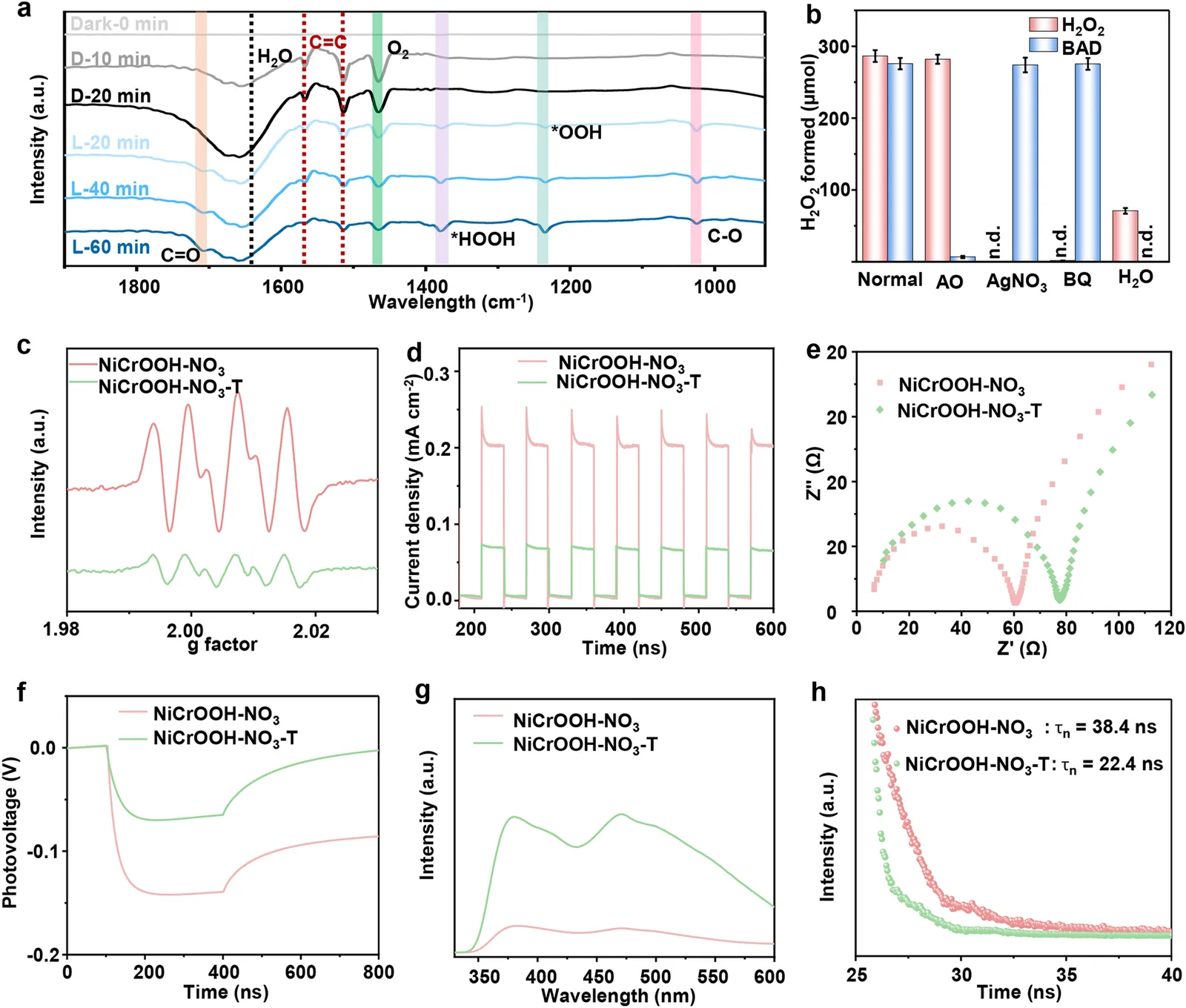
**a** In situ DRFTS spectra of NiCrOOH-NO_3_. **b** Effect of different reactive species scavengers on the photocatalytic H_2_O_2_ and BAD yield of NiCrOOH-NO_3_.** c** ESR signals of DMPO-·O_2_^−^ of NiCrOOH-NO_3_ and NiCrOOH-NO_3_-T. **d** Transient photocurrent responses, **e** EIS curves, **f** OCVD curves, **g** PL spectra and **h** TRPL spectra of NiCrOOH-NO_3_ and NiCrOOH-NO_3_-T

To track the reaction process of photocatalytic 2e^−^ ORR coupled with BAOR over NiCrOOH-NO_3_, in situ irradiated XPS (ISIXPS) and in situ diffuse reflectance infrared Fourier transform spectroscopy (DRIFTS) measurements were employed. The in situ XPS spectra of Ni 2*p*, Cr 2*p*, O 1*s*, and N 1*s* are shown in Fig. S23. Compared to the Ni 2*p* spectrum in the dark without O_2_, the Ni 2*p*_3/2_ peak of NiCrOOH-NO_3_ exhibits a positive shift of 0.33 eV after introducing O_2_ for 20 min, which may be attributed to the chemisorption of O_2_ on Ni sites. After 5 min of light irradiation, the binding energy of Ni 2*p*_3/2_ peak increases by 0.75 eV due to the electron depletion for photocatalytic 2e^−^ ORR. Different from Ni, the binding energies of Cr peaks are almost identical in the spectra recorded with and without O_2_, indicating that Cr is not the active site for O_2_ adsorption. For O 1*s*, the spectra before and after introducing O_2_ are also similar in darkness. Upon irradiation, an additional peak of M-OOH emerges at 532.30 eV, verifying the formation of the *OOH intermediate on the catalyst surface. Moreover, compared to the N 1*s* spectrum in darkness, the peak of intercalated NO₃⁻ in the spectrum under irradiation shows a negative shift of 0.21 eV, indicating an increased electron density of NO_3_^−^ with the accumulation of photogenerated electrons. The DRIFT spectrum in the dark (Fig. 3a) shows the peaks assigned to the bands of O–O bonding of O_2_ (1465 cm^−1^), H_2_O (1650 cm^−1^) [62], and the stretching vibrations of C=C (1490 and 1555 cm^−1^) and C–O (1027 cm^−1^) bonds in BA [36]. By applying light irradiation for 20 min, the peak intensity of the above four reactants is reduced with three new peaks of C=O stretching mode of BAD (1700 cm^−1^), *OOH (1378 cm^−1^) and *HOOH (1233 cm^−1^) generated [41, 45]. By prolonging the irradiation time to 40 and 60 min, the peak intensity of the intermediates and products is continuously elevated with further consumed raw materials, suggesting the occurrence of 2e^−^ ORR to H_2_O_2_ and BAOR to BAD.

Subsequently, a series of control experiments were conducted under varying reaction conditions. As displayed in Fig. 3b, the yield of BAD dramatically decreases when adding ammonium oxalate (AO, a hole scavenger) into the reaction system. By introducing AgNO_3_ (an electron scavenger), the H_2_O_2_ evolution is nearly terminated. These observations indicate that the production of H_2_O_2_ and BAD is mainly driven by the photoexcited electrons and holes, respectively [45]. The presence of p-benzoquinone (BQ), a scavenger of **·**O_2_^−^, also greatly impedes the H_2_O_2_ production, implying that the **·**O_2_^−^ radical is the vital intermediate for H_2_O_2_ formation [63], in accordance with the observations of in-situ DRIFTS spectra. The electron paramagnetic resonance (EPR) spectra collected by using 5,5-Dimethyl-1-pyrroline N oxide (DMPO) as a spin trap (Fig. 3c) show the characteristic signals of ·O_2_^−^ radicals under illumination, further supporting a two-step single-electron reduction mechanism with ·O_2_^−^ as reaction intermediate (O_2_ + e^−^  → ·O_2_^−^, ·O_2_^−^  + 2H^+^  + e^−^  → H_2_O_2_) for H_2_O_2_ production [38]. Additionally, the NiCrOOH-NO_3_ exhibits stronger signal intensities than NiCrOOH-NO_3_-T (Fig. 3c), corresponding to the enhanced activity of NiCrOOH-NO_3_. Moreover, the H_2_O_2_ yield is dramatically reduced in the absence of BA (Fig. 3b), suggesting the crucial role of BA in facilitating O_2_ to H_2_O_2_ conversion.

To understand the reasons of enhanced properties of NiCrOOH-NO_3_, the activity and selectivity of ORR over NiCrOOH-NO_3_ and NiCrOOH-NO_3_-T were investigated via rotating ring-disk electrode (RRDE) measurements [64]. As shown in Fig. S24, the linear sweep voltammetry (LSV) curves include the ORR current at the disk electrode (solid line) and the H_2_O_2_ oxidation current at the ring electrode (dashed line). Compared to NiCrOOH-NO_3_-T, NiCrOOH-NO_3_ delivers higher current densities of both ORR and H_2_O_2_ oxidation, showing a higher activity. Combining the disk and ring currents, the 2e^−^ ORR selectivity of NiCrOOH-NO_3_ and NiCrOOH-NO_3_-T was calculated to be 93.3 and 95.9% (Fig. S24b), respectively, favoring the 2e^−^ ORR pathway.

Afterward, photoelectrochemical and photoluminescence (PL) measurements were employed for determining the charge transfer efficiencies and dynamics. The photocurrent response profiles in Fig. 3d manifest higher photocurrent density of NiCrOOH-NO_3_ than NiCrOOH-NO_3_-T, indicating more efficient separation of photoinduced electron–hole pairs of NiCrOOH-NO_3_. The Nyquist plots (Fig. 3e) obtained by electrochemical impedance spectroscopy (EIS) test show that the arc radius of NiCrOOH-NO_3_ is smaller than that of NiCrOOH-NO_3_-T, revealing a lower charge transfer resistance and faster charge transfer rate. To further evaluate the kinetic characteristics of photogenerated charges, the open-circuit voltage decay (OCVD) curves of NiCrOOH-NO_3_ and NiCrOOH-NO_3_-T (Fig. 3f) were recorded [65]. Under light illumination, NiCrOOH-NO_3_ exhibits a higher open-circuit voltage than NiCrOOH-NO_3_-T. When the light is switched off, the slower photovoltage decay of NiCrOOH-NO_3_ suggests a longer lifetime of photoexcited carriers. Figure 3g displays the steady PL spectra. Compared to NiCrOOH-NO_3_-T, the PL intensity of NiCrOOH-NO_3_ is significantly reduced, indicating the suppressed recombination of photogenerated charge carriers. As a commonly used technique for investigating the electron–hole pair separation kinetics, time-resolved photoluminescence spectroscopy (TRPL) test was also performed. As shown in Fig. 3h, the fluorescence lifetime of NiCrOOH-NO_3_ is estimated to be 38.4 ns by double exponential fitting method, longer than that of NiCrOOH-NO_3_-T (22.4 ns), further verifying the improved charge separation ability of NiCrOOH-NO_3_.

**Fig. 4:**
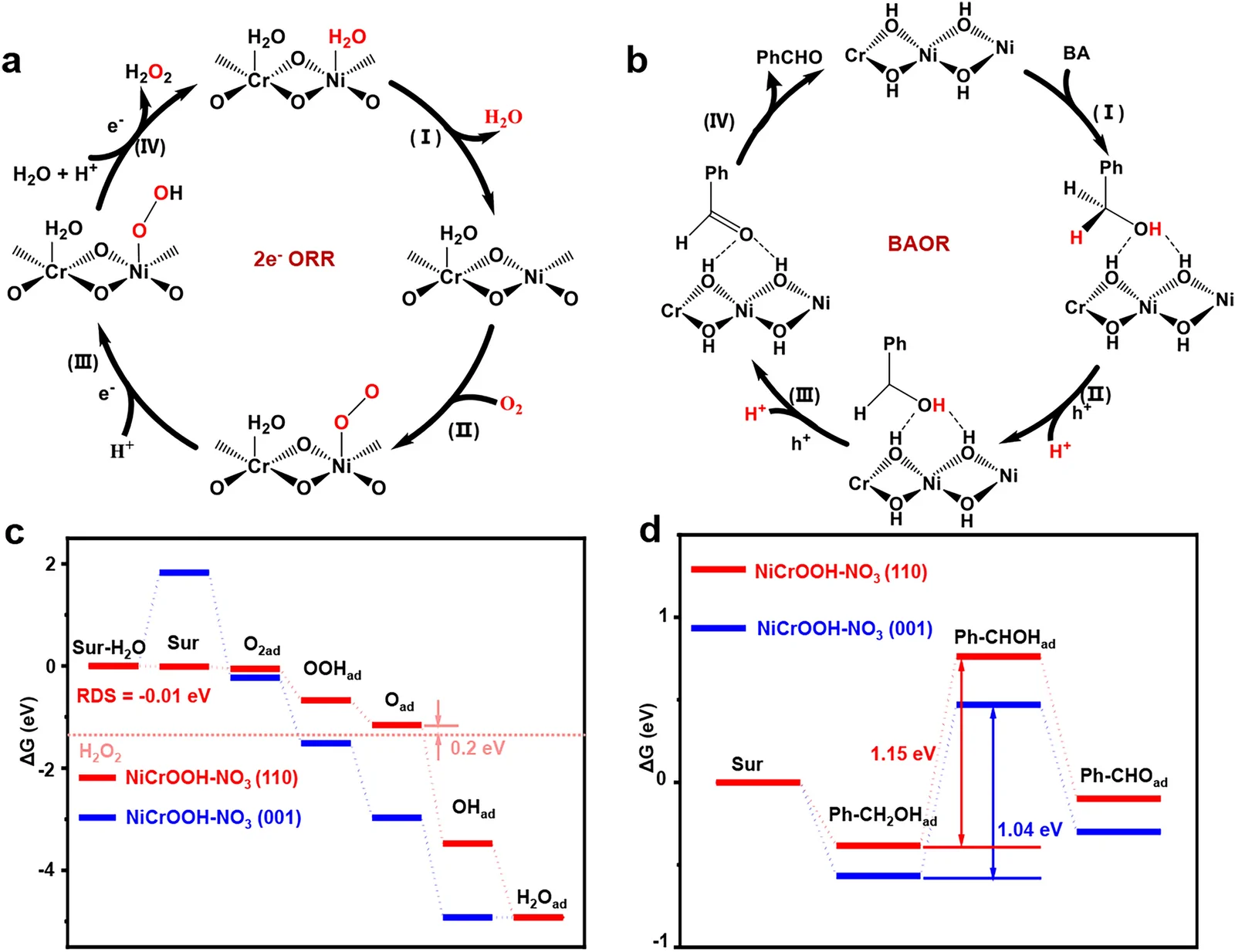
The proposed 2e^−^ ORR **a** and BAOR **b** catalytic cycle diagrams over NiCrOOH-NO_3_. The reaction profiles of **c** the ORR and **d** BAOR on NiCrOOH-NO_3_

To provide understandings of the photocatalyst structure-performance relationship, comprehensive density functional theory (DFT) calculations were conducted. Firstly, we aimed to understand the impact of facet exposure on the photocatalytic performance. The reaction profiles and optimized intermediates structures of the ORR and BAOR on the (110) and (001) surfaces of NiCrOOH-NO_3_ are shown in Figs. 4 and S25. For 2e^−^ ORR, step I involves the leaving of a surface coordination *H_2_O and *OH on (110) and (001) surfaces, respectively, to create an unsaturated Ni^*^ site (Fig. 4a). Subsequently, O_2_ is adsorbed on the exposed metal sites to form O_2ad_ (step II) [66]. Then, the reduction in O_2ad_ to OOH_ad_ by photo-generated electrons (step III) and further reduction in OOH_ad_ generate H_2_O_2_ (step IV) to complete a 2e^−^ ORR cycle. Alternatively, OOH_ad_ can be reduced to O_ad_ and OH_ad_, forming H_2_O in a competitive 4e^−^ ORR pathway [67]. From the free energy change (ΔG) in each step of the ORR process (Fig. 4c), step I is the rate-determining step (RDS). Moreover, the ΔG of Step I over the (110) facet of NiCrOOH-NO_3_ is − 0.01 eV, lower than that for the (001) facet (1.83 eV) with exposure of hydroxyl groups, indicating that the (110) facet is a preferred site for the ORR process. In addition, the ΔG of OOH_ad_ → O_ad_ over (110) facet is 0.2 eV higher than the ΔG of OOH_ad_ → H_2_O_2_. In contrast, the ΔG of OOH_ad_ → O_ad_ is negative while the ΔG of OOH_ad_ → H_2_O_2_ is positive over the (001) facet. The calculation results indicate that the 2e^−^ ORR pathway is more favorable on the (110) facet than the (001) facet.

For a BAOR cycle, BA is adsorbed on the catalyst in step I (Fig. 4b). The formed Ph-CH_2_OH_ad_ is preferentially oxidized to Ph-CHOH_ad_ by the holes (h^+^) in step II. Then, Ph-CHOH_ad_ is further oxidized to Ph-CHO_ad_ in step III before the release of the final Ph-CHO product in step IV. It is noted that the ΔG of the first three steps is consistently lower over the (001) surface of NiCrOOH-NO_3_ than the (110) surface (Fig. 4d), mainly due to the hydrogen bonding interactions between the hydroxyl groups on the (001) facet of the catalyst and the oxygen-containing groups in Ph-CH_2_OH_ad_, Ph-CHOH_ad_ and Ph-CHO_ad_ intermediates (Fig. 4d), suggesting that the (001) facets are kinetically favorable for BA oxidation.

The above results indicate that the photo-generated electrons are mainly located on the (110) facet for reduction reactions while the holes on the (001) facet for oxidation reactions. To further support the facet-dependent charge separation, Kelvin probe force microscopy (KPFM) analysis was performed under dark and illumination conditions. Figure S26 shows the topographic image of NiCrOOH-NO₃ nanosheets. A statistical histogram is presented by measuring the light-induced potential difference of eight independent nanosheets (Fig. S26a-c). Elevated surface potential of (001) facet in NiCrOOH-NO_3_ is observed under illumination (average 31.20 mV) compared to that in dark (average 11.95 mV), suggesting the accumulation of photogenerated holes on the (001) facet. Moreover, from the production rates of H_2_O_2_ and BAD, the ratio of consumed electrons and holes is estimated to be 1/0.96 for NiCrOOH-NO_3_, closer to 1/1 than that for NiCrOOH-NO_3_-T (1/0.67). This observation is consistent with the simulation results, suggesting that the higher exposure of {001} planes in NiCrOOH-NO_3_ than NiCrOOH-NO_3_-T facilitates the hole consumption and BAOR. Thus, the spatially separated (110) and (001) facets with optimized facet exposure ratio enables the efficient utilization of photogenerated electrons and holes with enhanced photocatalytic performance.

**Fig. 5:**
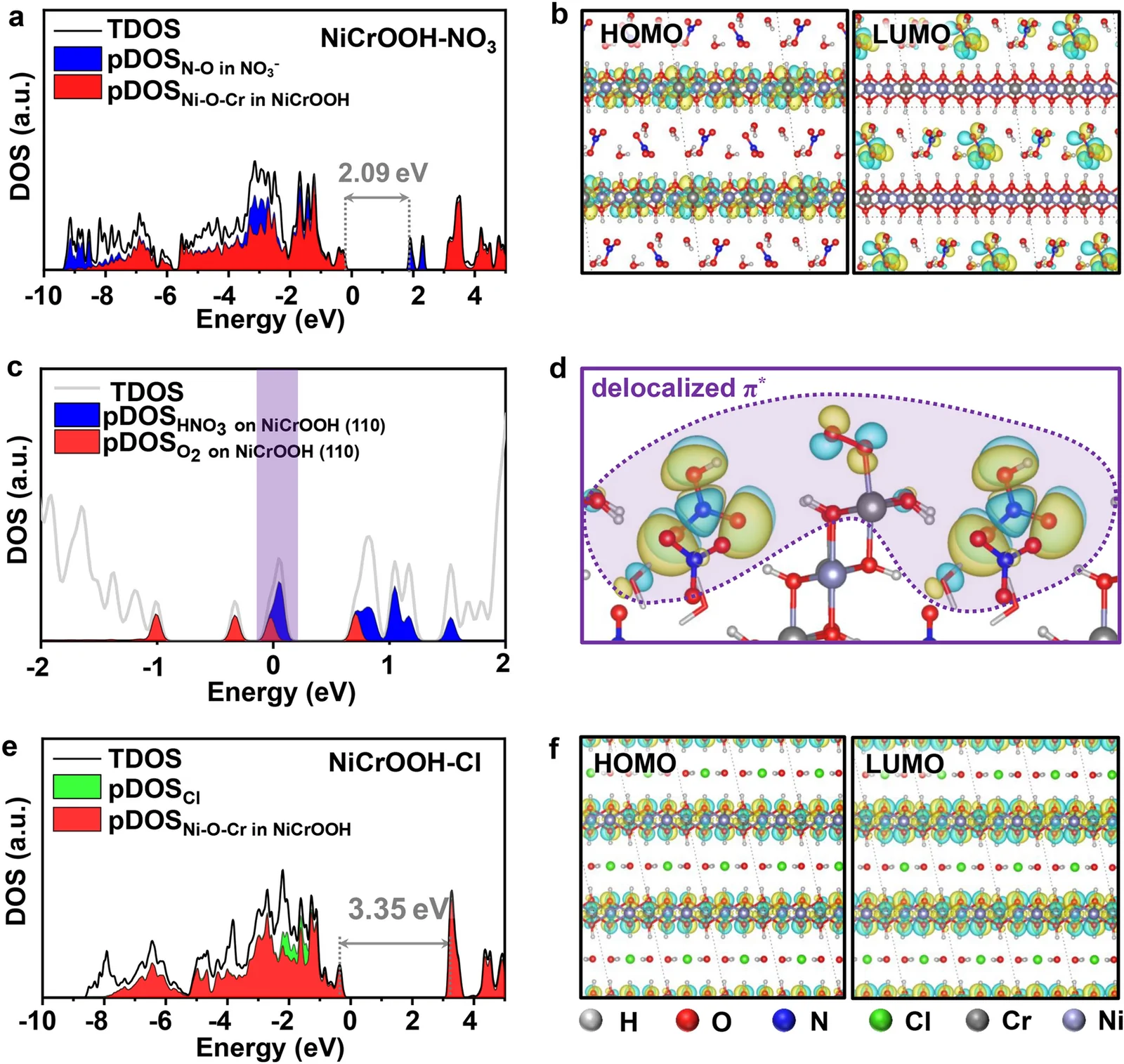
**a** TDOS and pDOS of NO_3_⁻ and Ni–O–Cr layer for NiCrOOH-NO_3_. **b** HOMO and LUMO isosurfaces of NiCrOOH-NO_3_. **c** TDOS and pDOS of NO_3_⁻ and adsorbed O_2_ on (110) surface of NiCrOOH-NO_3_ in the presence of additional H^+^  + e⁻ pair. **d** Wavefunction isosurfaces of the states at the VBM of NiCrOOH-NO_3_ in the presence of adsorbed O_2_ and additional H^+^  + e⁻ pair. **e** TDOS and pDOS of Cl⁻ and Ni–O–Cr layer for NiCrOOH-Cl. **f** HOMO and LUMO isosurfaces of NiCrOOH-Cl. In the wavefunction isosurfaces, the yellow and green isosurfaces represent the positive and negative regions of the wave function, respectively

Secondly, to elucidate the structural basis of the improved 2e^−^ ORR and BAOR performance of NiCrOOH-NO_3_, the profiles of total density of states (TDOS) and projected density of states (PDOS) are illustrated in Fig. 5a, and the highest occupied molecular orbital (HOMO)-lowest unoccupied molecular orbital (LUMO) analysis in Fig. 5b. For NiCrOOH-NO_3_, the valance band maximum (VBM) is mainly dictated by the Ni–O–Cr orbital (indicated in red), while the conduction band maximum (CBM) is predominantly contributed by the N–O orbitals of NO_3_^−^ (blue). Thus, the holes are in the Ni–O–Cr units (HOMO) while the excited electrons are localized in antibonding π^*^ orbitals of interlayer NO_3_^−^ (LUMO). The bandgap of NiCrOOH-NO_3_ is calculated to be 2.09 eV, close to the experimental findings (Fig. S7b).

Next, the DOS analysis in the presence of both adsorbed O_2_ molecule and additional H^+^ + e^−^ pair was studied in the photocatalytic process (Fig. 5c). Compared to the original NiCrOOH-NO_3_, the introduction of adsorbed O_2_ molecule and additional H^+^ + e⁻ results in a significant change of the DOS profile. The VBM (shown in purple) is shifted to the previously unoccupied π* orbital of NO_3_⁻ (blue) that overlaps with the bonding peak of π* orbital of adsorbed O_2_ molecule (red). The wavefunction isosurfaces of the VBM state (Fig. 5d) illustrate that the adsorbed oxygen molecules can acquire electrons through the coupled π* orbitals, thereby facilitating subsequent reduction reactions. These results suggest the crucial role of interlayer NO_3_^−^ in electron–hole generation upon photoexcitation and intramolecular electron transfer in manipulating the photocatalytic performance.

For comparison, a control sample NiCrOOH-Cl with interlayer Cl^−^ was prepared and characterized (see details in Figs. S27 and S28) [68]. As shown in Figs. S27 and S28; Table S3, NiCrOOH-Cl possesses similar morphology, crystal structure and specific surface area with NiCrOH-NO_3_. The photochemical results show that H_2_O_2_ (11.1 mmol g^−1^ h^−1^) and BAD (10.5 mmol g^−1^ h^−1^) yields of NiCrOOH-Cl are both inferior than NiCrOOH-NO_3_ (Fig. S29). Besides, the 2e^−^ ORR selectivity of NiCrOOH-Cl was determined as 93.0% (Fig. S30), close to that of NiCrOOH-NO_3_ (93.3%). The lower photocurrent response and fluorescence lifetime, larger impedance radius and higher fluorescence intensity (Fig. S31) further indicate that the electron hole separation capacity of NiCrOOH-Cl is lower than that of NiCrOOH-NO_3_. Different from NiCrOOH-NO_3_, both the VBM and CBM of NiCrOOH-Cl are contributed by the Ni–O–Cr orbitals (Fig. 5e). From the pDOS profile (Fig. 5f), both the photoexcited holes and electrons are located in the Ni–O–Cr units, leading to insufficient charge separation. The O_2_ adsorption on NiCrOOH-Cl follows a side-on mode, the same as that on NiCrOOH-NO_3_ (Fig. S32). These results further indicate the unique role of interlayer NO_3_^−^ in not only facilitating the ORR process but also promoting electron–hole separation.

## Conclusions

In summary, ultrathin NiCrOOH-NO_3_ nanosheets have been designed as an efficient photocatalyst for 2e^−^ ORR integrated with BAOR. Systematical investigations reveal the synergistic roles of facet engineering and NO_3_^−^ intercalation in improving photocatalytic performance. The spatial separation of active sites for 2e^−^ ORR on (110) planes and BAOR on (001) planes facilitate the directional migration of photogenerated electrons and holes, promoting charge separation. The interlayered NO_3_^−^ ions favor the electron–hole pair generation and promote the electron transfer to oxygen intermediate via intermolecular electron delocalization. As a result, the NiCrOOH-NO₃ photocatalyst exhibits record-high performance with a H₂O₂ production rate of 28.7 mmol g⁻^1^ h⁻^1^ and a SCC efficiency of 3.92%. Our findings have paved the way for the development of advanced LDH structures and high-performance 2e^−^ ORR photocatalysts.

## Supplementary Information

Below is the link to the electronic supplementary material.

Supplementary file1 (DOCX 3994 KB)
