# Supplementary material for: Photocatalytic H2O2 Production over Ultrathin Layered Double Hydroxide with 3.92% Solar-to-H2O2 Efficiency
Source: Nanomicro Lett. 2026 Jan 12;18:192. doi: 10.1007/s40820-025-02044-0 (PMC12791099; doi:10.1007/s40820-025-02044-0)
Supplement: Supplementary file 1 — Supplementary file1 (DOCX 3994 KB) [file 40820_2025_2044_MOESM1_ESM.docx]

Supporting Information for

**Photocatalytic H₂O₂ Production over Ultrathin Layered Double Hydroxide with 3.92% Solar-to-H₂O₂ Efficiency**

Yamin Xi^1^ , Zechun Lu^3^ , Tong Bao^1^ , Yingying Zou^1^ , Chaoqi Zhang^1^ , Chunhong Xia^1^ , Guangfeng Wei^3*^ , Chengzhong Yu^1,2,4*^ , and Chao Liu^1,2,5*^

^1^ School of Chemistry and Molecular Engineering, East China Normal University, Shanghai 200241, P. R. China

^2^ State Key Laboratory of Petroleum Molecular and Process Engineering, SKLPMPE, East China Normal University, Shanghai 200241, P. R. China

^3^ Shanghai Key Laboratory of Chemical Assessment and Sustainability, School of Chemical, Science and Engineering, Tongji University, Shanghai, 200092, P. R. China

^4^ Australian Institute for Bioengineering and Nanotechnology, The University of Queensland, Brisbane, QLD 4072, Australia

^5^ Shanghai Frontiers Science Center of Molecule Intelligent Syntheses, School of Chemistry and Molecular Engineering, East China Normal University, Shanghai 200241, P. R. China

*Corresponding authors. E-mail: [weigf@tongji.edu.cn](mailto:weigf@tongji.edu.cn) (Guangfeng Wei); [czyu@chem.ecnu.edu.cn](mailto:czyu@chem.ecnu.edu.cn) or [c.yu@uq.edu.au](mailto:c.yu@uq.edu.au) (Chengzhong Yu); [cliu@chem.ecnu.edu.cn](mailto:cliu@chem.ecnu.edu.cn) (Chao Liu)

**S1 Characterization**

Transmission electron microscopy (TEM) and high-resolution transmission electron microscopy (HRTEM) images were collected on a JEM-2100 F (JEOL, Japan) with an acceleration voltage of 200 kV. The element analysis was conducted on a field-emission scanning electron microscope (FIB, Helios G4 UX, FEI Inc. USA) equipped with an X-ray energy dispersive spectrometer (EDS: X-Max 150T, Oxford, UK). X-ray photoelectron spectroscopy (XPS) measurement was carried out on a Thermo ESCALAB 250 using an Al Kα radiation and C 1s (284.8 eV) as a reference to correct the binding energy (the resolution is 0.1 eV). XRD patterns were recorded using a Bruker D8 advanced X-ray diffractometer with Cu Kα radiation (λ = 0.154 nm). UV/vis diffuse reflectance spectra were collected on a Lambda950 spectrophotometer. BaSO_4_ was used as the reflectance standard. Room temperature photoluminescence (PL) spectra were recorded on an Edinburgh FS5 spectrofluorometer in the range of 390 − 600 nm. Time-resolved photoluminescence (TRPL) spectra were taken on a FSL980 transient fluorescence spectrometer. Electron spin resonance (ESR) spectra were recorded on a Bruker A300 ESR spectrometer. Thermogravimetric analysis (TGA) was conducted on a Mettler-Toledo TGA/SDTA851e equipment. Chemical analyses for C, H, N, and O elements were conducted on an ELEMENTAR Vario micro (Germany) element analyzer. The Ni and Cr contents in the samples were determined by inductively coupled plasma atomic emission spectrometry (ICP-AES), carried on an Optima 7000DV spectrometer.

**S2 Photocatalytic measurements**

In a typical reaction setup, 10 mg of photocatalysts were dispersed in the mixture solution of H_2_O (20 mL) and BA (4 mL). The resultant mixture was sonicated and bubbled with oxygen for 30 min in the dark. Subsequently, the photoreaction was initiated by irradiation of a 300 W xenon lamp (PLS-SXE300D/300DUV, Perfect Light). After photocatalytic reactions, the supernatant was isolated by centrifugation, then filtrated with a millipore filter (0.45 µm) to remove the photocatalyst. The H_2_O_2_ concentration was determined by iodometry. Typically, 50 μL of treated reaction solution was added to 1 mL of mixture solution of 0.05 M C_8_H_5_KO_4_ and 0.2 M KI. Under acidic condition, the H_2_O_2_ molecules will react with I^−^ to generate I_3_^−^. After reaction for 30 min, the amount of I_3_^−^ was measured by a UV spectrophotometer at 350 nm, which was further used to quantify the generated H_2_O_2_. The concentration of benzaldehyde generated in the reaction was determined by high-performance liquid chromatography (HPLC) (LC20AD, Shimadzu, Japan) using a UV-vis detector (SPD-20A, Shimadzu, Japan) and a 5 μm C18 column. 100 μL of the reaction solution was injected, and the mobile phase, comprising a methanol-phosphoric acid aqueous solution (3:7, v/v), was delivered at a flow rate of 1 mL/min. The detection wavelength was set as 254 nm.

**S3 H_2_O_2_ quantification by** **ferrous ion oxidation xylenol orange colorimetry method**

The H_2_O_2_ concentration was further determined using a ferrous ion oxidation xylenol orange colorimetry method. Typically, a ferrous ion oxidation xylenol orange (FOX) solution was prepared by dissolving Fe(NH_4_)_2_(SO_4_)_2_·6H_2_O (19.61 mg), D-sorbitol (3.644 mg), and xylenol orange (XO) (14.333 mg) in deionized water (200 mL) added with ethanol (2 mL) and H_2_SO_4_ (98%, 272 μL). Subsequently, 50 μL of the obtained H_2_O_2_ solution (diluted if needed) was mixed with the pre prepared FOX solution. The concentration of H_2_O_2_ was quantified by monitoring the characteristic absorption peak at 560 nm via UV-Vis spectroscopy according to the calibration curve (Fig. S11).

**S4 Apparent quantum yield (AQY) and solar-to-chemical conversion (SCC) efficiency**

Apparent quantum efficiency (AQY) was measured by illuminating the suspension of NiCrOOH-NO_3_ with monochromated light (λ = 365, 400, 420, 500, 550, 600, and 650 nm). The photocatalytic reactions were carried out in a circular quartz reactor with an inner diameter of 4 cm. The light source illuminated the reactor from the top. The optical path length through the reaction suspension (24 mL) was 1 cm. The full-width at half-maximum of each bandpass filter was approximately ±15 nm. In addition, an [optical power](https://www.sciencedirect.com/topics/materials-science/optical-power) meter (PL-MW2000, Beijing Perfectlight Technology) was utilized to detect the light intensity. AQY for H_2_O_2_ production was calculated using the following equation:

AQY (H_2_O_2_) = ([the number of evolved H_2_O_2_ molecules × 2]) / (photon number) × 100

AQY (%)=N_electron_/N_photon_ = 2N(H_2_O_2_)/[(I×A×t)/(E_photon_×N_A_)],

where N(H_2_O_2_) represents the amount of H_2_O_2_; I is the incident light intensity; A is the illumination area (3.14 cm^2^); t is the illumination time; N_A_ is Avogadro’s constant. Besides, E_photon_ refers to the average single photon energy, which is calculated using the equation [S1, S2]:

E_photon_ = hc/λ,

where h is the Planck constant, c is the speed of light, and λ is the wavelength.

The absorbed-photon-to-chemical efficiency (APCE) was calculated to evaluate the intrinsic efficiency by using the following equation [S3]:

APCE (%) = AQY (%) / A,

where AQY is calculated via incident photons and H_2_O_2_ yield, and A is the absorption of photocatalyst solution.

To determine the SCC efficiency, 0.6 g/L of catalyst was dispersed in water and BA solution (volume ratio of 5/1) with O_2_ bubbling at 333  K (Fig. S15). An AM1.5G solar simulator was used as light source. The photocatalytic reaction was conducted for 1 h under stirring. To investigate the effects of optical depth, reaction solutions with various total volumes from 60 to 150 mL were used with the stirring rate settled as 800 rpm. Besides, the effect of stirring rate was also explored by using different stirring rates from 0 to 800 rpm in 120 mL reaction solution (Figs. S16, S17). The SCC efficiency was determined using:

$$\text{SCC efficiency (\%) = }\frac{\text{[}\Delta G\text{ for H}_{2}\text{O}_{2}\text{ generation (J mol}^{-1}\text{)] }\times\text{ [H}_{2}\text{O}_{2}\text{ formed (mol)]}}{\text{[total input energy (W)] }\times\text{ [reaction time (s)]}}\text{ }\times\text{ 100}$$

The free energy for H_2_O_2_ formation is 117 kJ mol^–1^, the irradiance of the AM1.5 global spectrum (300-2500 nm) is 1000 W m^–2^ and the irradiated area is 3.14 × 10^–4^  m^2^, giving a total input energy of 0.314  W.

**S5 Isotope labelling experiments**

5 mg of catalysts and 1 mL of H_2_O were put in a sealed quartz vial (5 mL), followed by ultrasonication for 5 min. Pure Ar was bubbled into the resultant suspension for 30 min in the dark. Then, 5 mL of ^18^O_2_ was injected and the reactor was irradiated with a 300 W xenon lamp for 5 h. After removing the remaining ^18^O_2_ gas, excessive MnO_2_ aqueous solution (Ar saturation) was added into the reactor to convert H_2_O_2_ into O_2_, and the gas products were eventually analyzed by gas chromatography-mass spectrometry (GCMS-QP2010 SE, SHIMADZU).

**S6 Photoelectrochemical test**

Photoelectrochemical measurements were performed on a Chenhua CHI 760E electrochemical workstation by a standard three-electrode cell system in 0.1 M Na_2_SO_4_ electrolyte. The Pt wire, Ag/AgCl, and photocatalyst modified indium-doped tin oxide (ITO) were used as counter, reference and working electrodes, respectively. The working electrode was prepared as follows: 10 mg of catalysts was first mixed with 30 µL of 10% Nafion solution to make a slurry. Afterward, the slurry was pipetted onto a piece of ITO glass (effective area: 1 cm^2^), and then dried at 55 °C overnight. The photocurrent was recorded under simulated sunlight using a 300 W xenon lamp as light source. Electrochemical impedance spectroscopy (EIS) curves were obtained in a frequency range from 0.01 Hz to 1000 kHz under irradiation. The transient open-circuit voltage decay (OCVD) measurements were taken for a total of 800 s, and the visible light was switched on and off after 100 and 400 s from the start, respectively. The average lifetimes of the photogenerated carriers (*τ_n_*) were then estimated from the open-circuit voltage (V_oc_) decay according to the following Equation:

$$\tau_{n}=-\frac{k_{B}T}{q}\left(\frac{dV_{oc}}{dt}\right)^{-1}$$

where *τ_n_* represents the average lifetime, *V_oc_* is open-circuit voltage, *k_B_* is the Boltzmann constant, *T* is the temperature (in Kelvin), and *q* is the unsigned charge of an electron.

**S7 Computational methods**

Spin-polarized density functional theory (DFT) calculations were performed using LASP (www.lasphub.com) program [S4] contained VASP 6.2.1 packages [S5] with projected augmented wave (PAW) pseudo-potentials [S6, S7]. The exchange-correlation energy was treated based on the generalized gradient approximation (GGA) by using Perdew Burke–Ernzerhof (PBE) functional [S8]. The DFT+U method was used to describe the Cr and Ni 3d electrons following previous studies [S9-S11]. The U_eff_ values of Cr 3d and Ni 3d are 3.2 and 3.8 eV, respectively. The plane-wave cutoff energy was set as 450 eV. The DFT-D3(BJ) method of Grimme [S12, S13] was employed to describe long range VDW interactions. The Monkhorst–Pack scheme with a k-point separation length of 0.05 Å^−1^ was utilized for sampling the first Brillion zone [14]. To correct the zero-point energy for reaction profiles, the vibrational frequency calculations were performed via the finite-difference approach. The solvation effects were considered by VASPsol package [S15, S16]. All atoms were fully relaxed in the calculations. The Quasi-Newton l-BFGS method was used for geometry relaxation until the maximal force on each degree of freedom less than 0.05 eV/Å. To derive the free energy reaction profiles, we followed the same approach as our previous work [S17]. The standard thermodynamic data [S18] was utilized to acquire the temperature and pressure contributions. In this work, the structure model was obtained via optimizing the experimental crystal structures. For example, the optimized lattice parameters of NiCrOOH-NO_3_ are 9.20 Å × 5.33 Å × 9.33 Å with α=88.3°, β=96.8°, γ=90.5°, and 9.22 Å × 5.29 Å × 8.79 Å with α=93.2°, β=77.3°, γ=89.9°, respectively. The optimized lattice parameters of NiCrOOH-Cl are 5.41 Å × 5.56 Å × 8.04 Å with α=103.9°, β=96.7°, γ=60.9°.


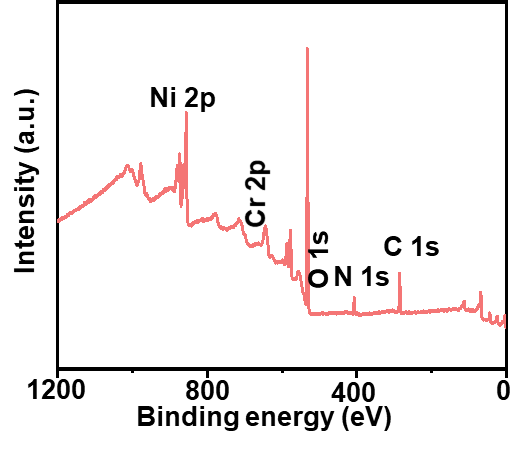


**Fig. S1** XPS survey spectrum of NiCrOOH-NO_3_


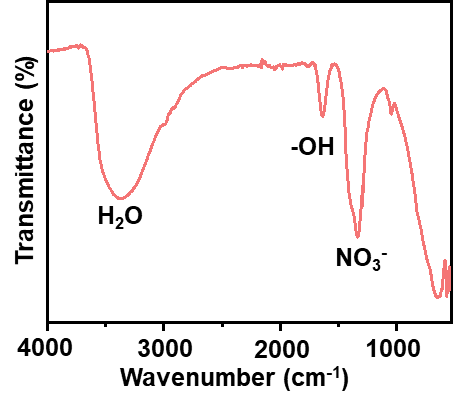


**Fig. S2** FTIR spectrum of NiCrOOH-NO_3_


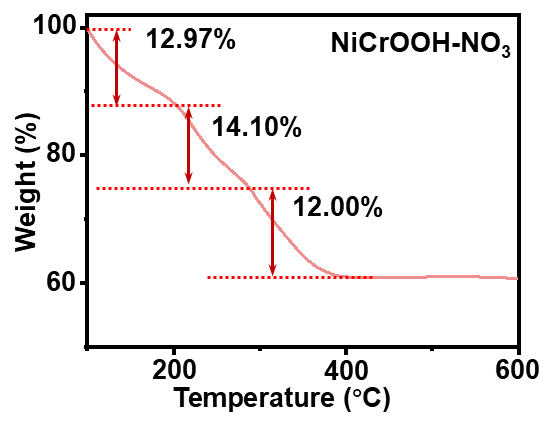


**Fig. S3** TGA curve of NiCrOOH-NO_3_


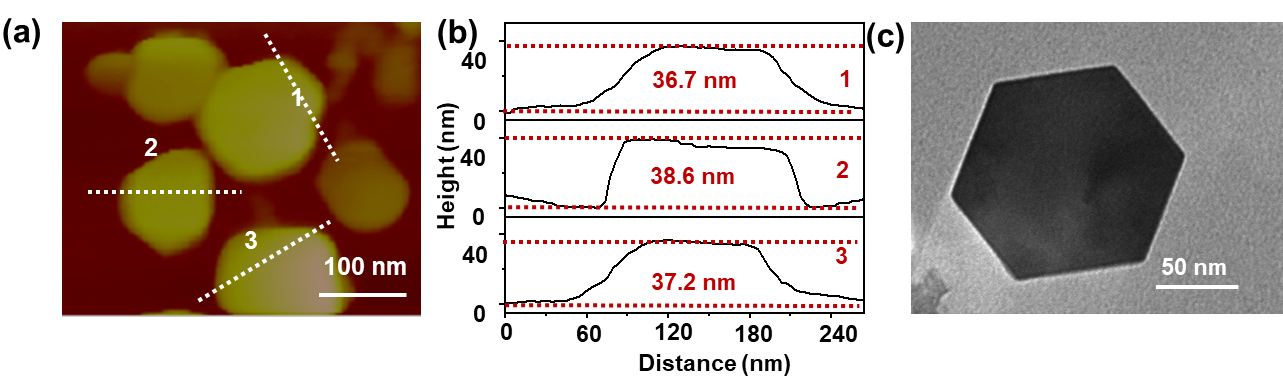


**Fig. S4** (**a**) AFM image and (**b**) height profiles of NiCrOOH-NO_3_-T nanosheets, (**c**) TEM image


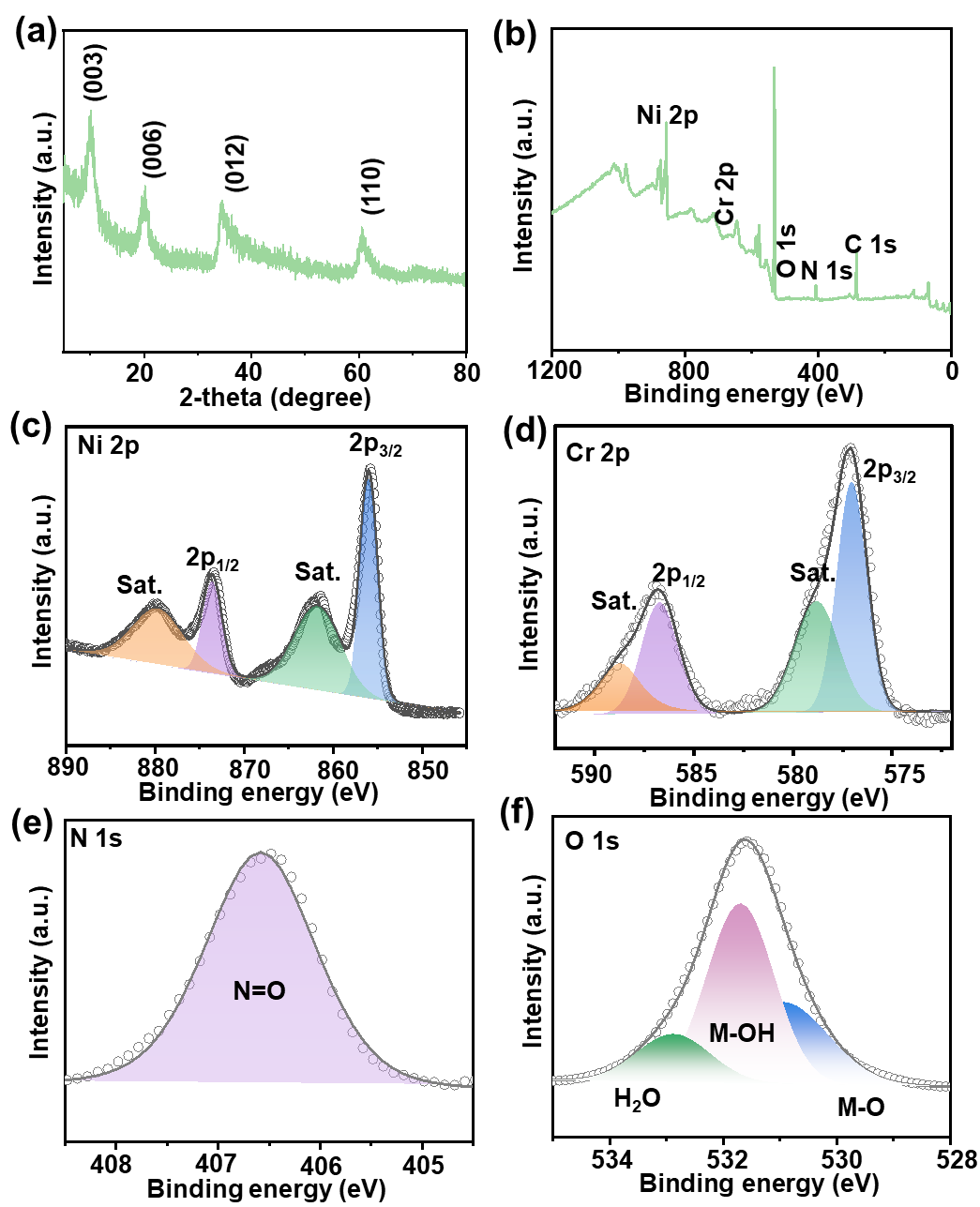


**Fig. S5** (**a**) XRD pattern, (**b**) XPS survey spectrum, high-resolution XPS spectra of (**c**) Ni 2p, (**d**) Cr 2p, (**e**) N 1s, (**f**) O 1s of NiCrOOH-NO_3_-T

**
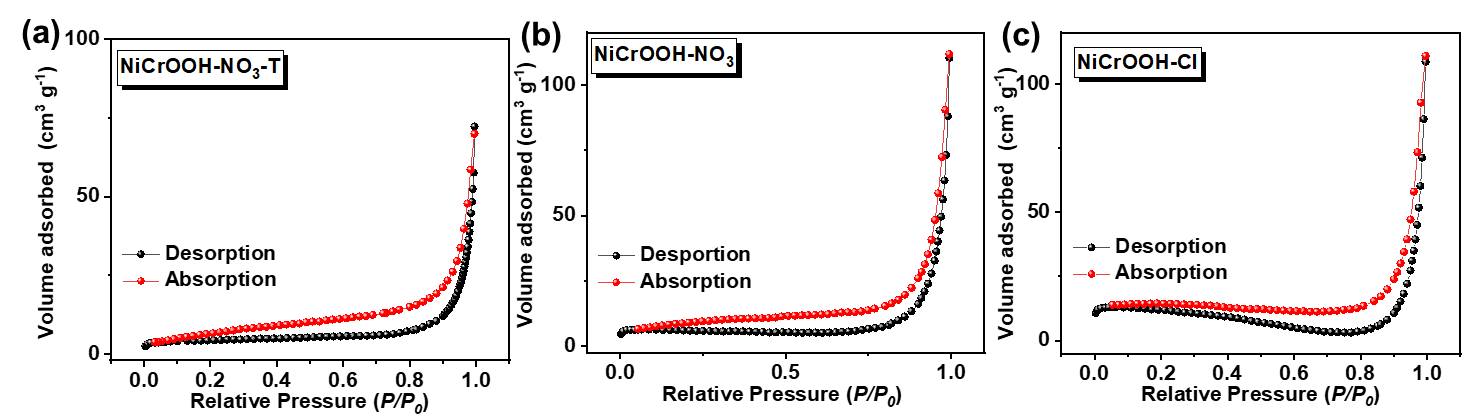
**

**Fig. S6** Nitrogen adsorption-desorption isotherms of NiCrOOH-NO_3_-T, NiCrOOH-NO_3_ and NiCrOOH-Cl


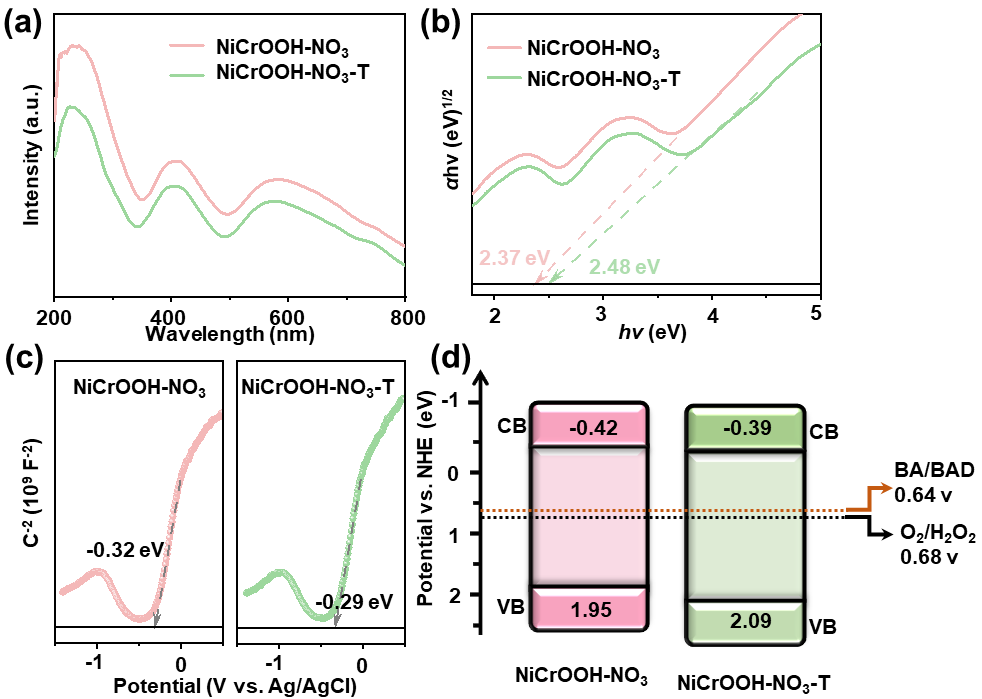


**Fig. S7** (**a**) UV–vis DRS profiles of NiCrOOH-NO_3_ and NiCrOOH-NO_3_-T, (**b**) Tauc plots and (**c**) Mott-Schottky plots, and (**d**) band structures of NiCrOOH-NO_3_ and NiCrOOH-NO_3_-T

**Fig. S8** Light irradiation spectrum of the xenon lamp (320 nm < λ < 780 nm)


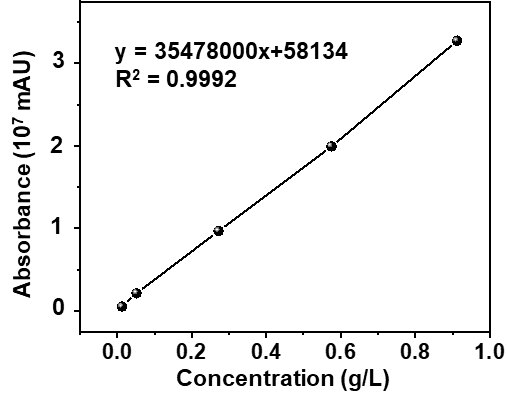


**Fig. S9** Standard curve between concentration and absorbance of benzaldehyde


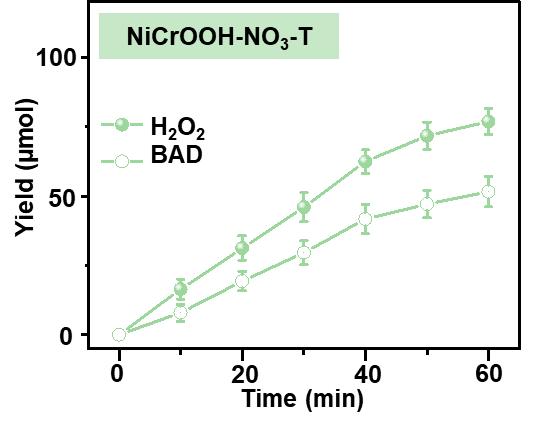


**Fig. S10** Time course of H_2_O_2_ and BAD contents during the photocatalytic reaction over NiCrOOH-NO_3_-T

**
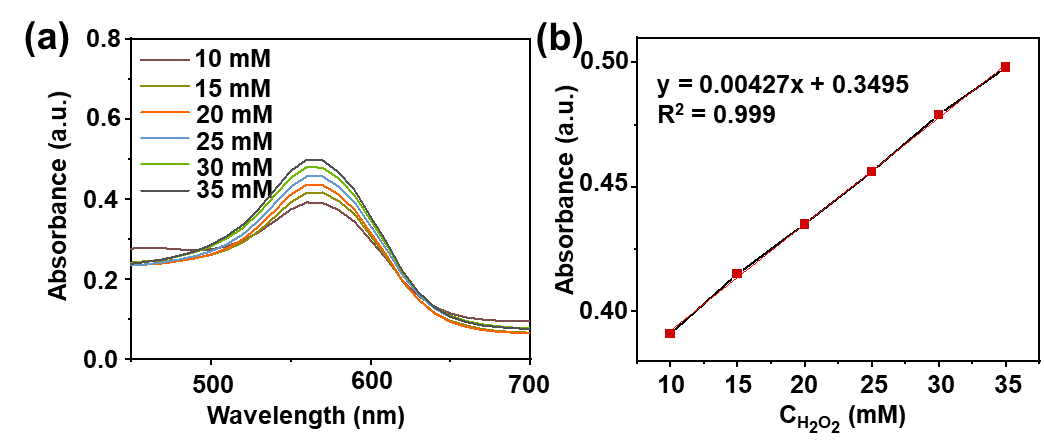
**

**Fig. S11** (**a**) UV-vis absorption spectra and (**b**) corresponding standard curve for H_2_O_2_ quantification by ferrous ion oxidation xylenol orange colorimetry

**
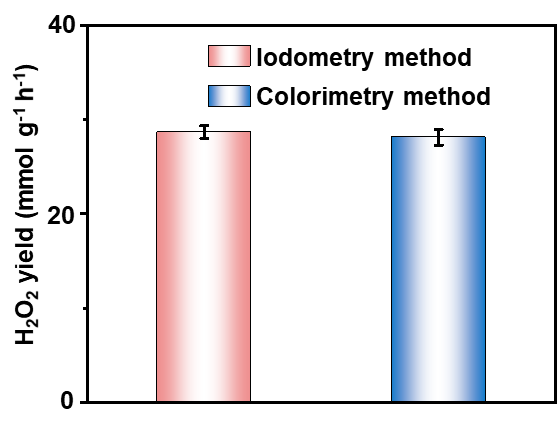
**

**Fig. S12** H_2_O_2_ yield rates determined by iodometry and colorimetry method


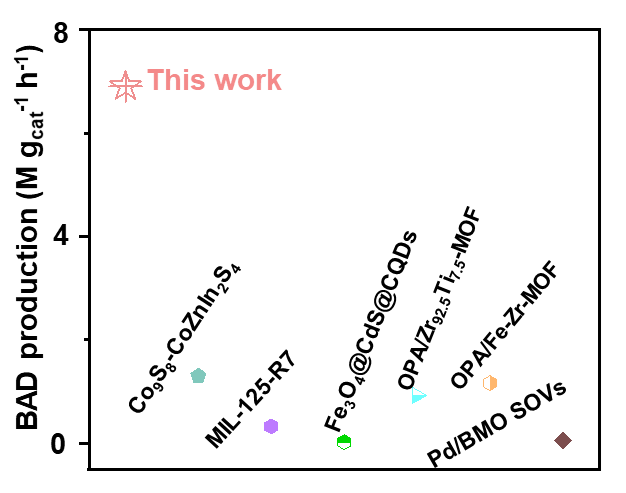


**Fig. S13** Comparison of BAD production with recently reported photocatalysts


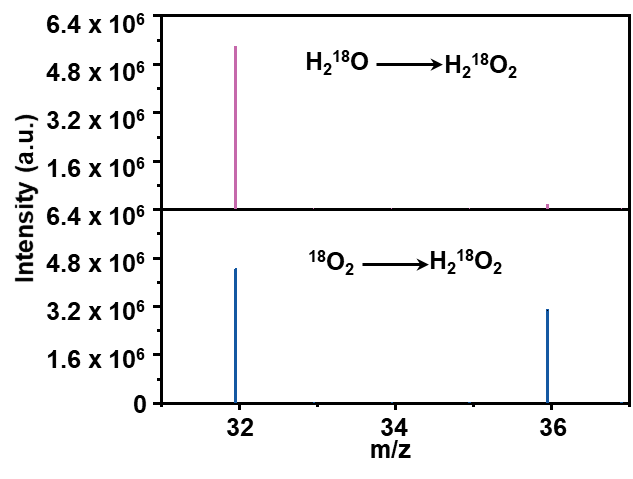


**Fig. S14** GC-MS spectra of O_2_ products generated from H_2_O_2_ decomposition in the isotope labelling experiment


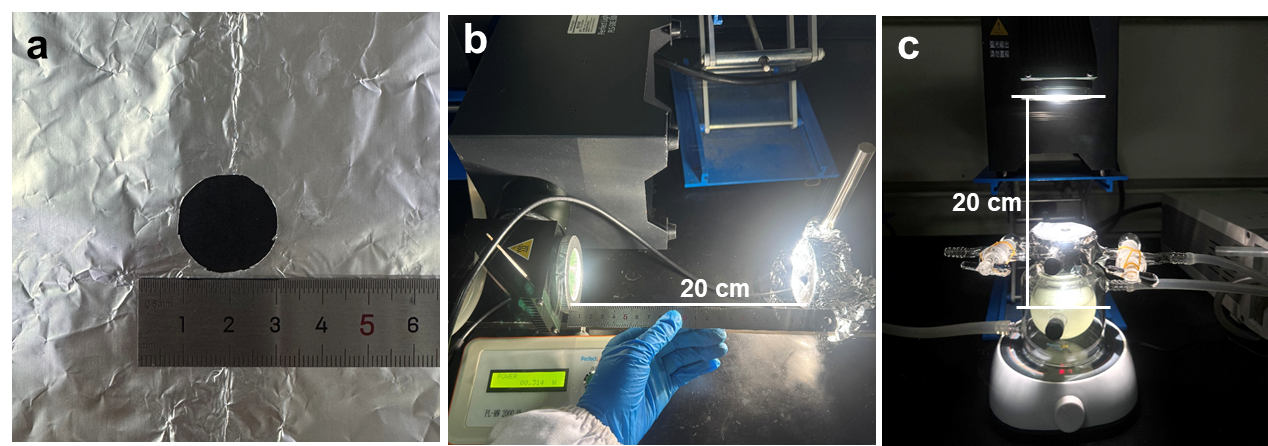


**Fig. S15** Image of (**a**) a circular hole with a radius of 1 cm in the aluminum foil sheet, (**b**) light intensity measurement via the Si photodiode, (**c**) reaction system for measuring SCC efficiency of NiCrOOH-NO_3_


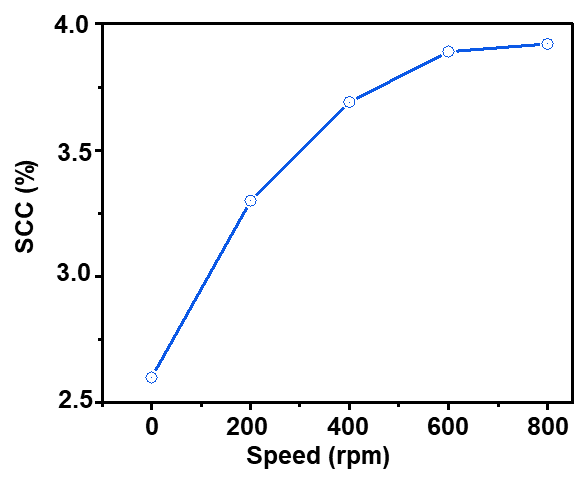


**Fig. S16** SCC efficiency at different rotational speeds


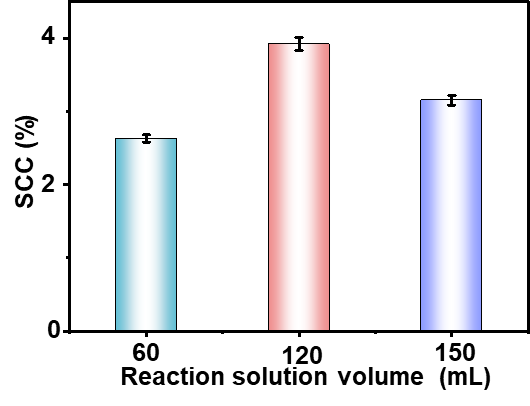


**Fig. S17** SCC efficiency of NiCrOOH-NO_3_ with different volumes of reaction solution

**
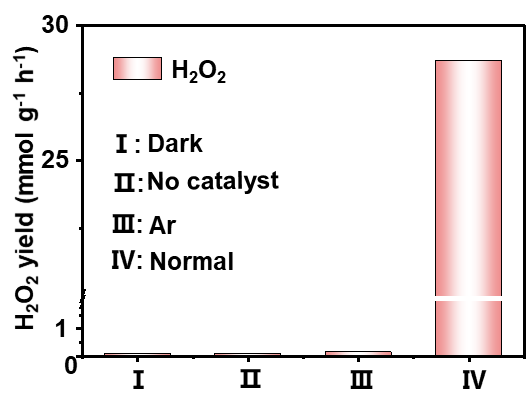
**

**Fig. S18** Photocatalytic H_2_O_2_ production over NiCrOOH-NO_3_ under different conditions


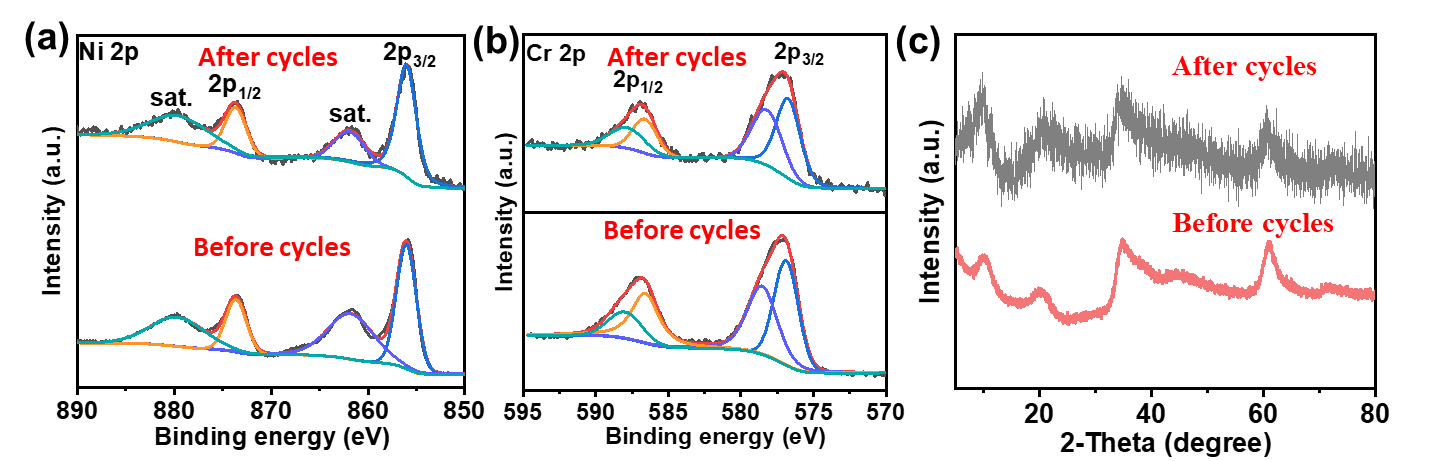


**Fig. S19** (**a**) Ni 2p, (**b**) Cr 2p XPS spectra and (**c**) XRD pattern of NiCrOOH-NO_3_ after cycling test

**
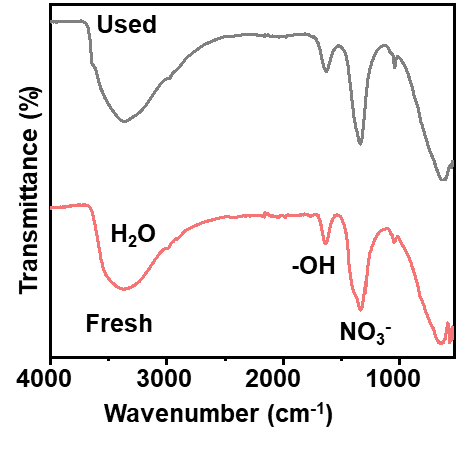
**

**Fig. S20** FTIR spectra of NiCrOOH-NO_3_ before and after cycling test


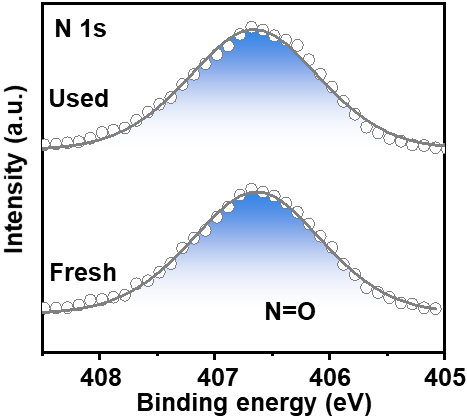


**Fig. S21** N 1s XPS spectra of NiCrOOH-NO_3_ before and after cycling test


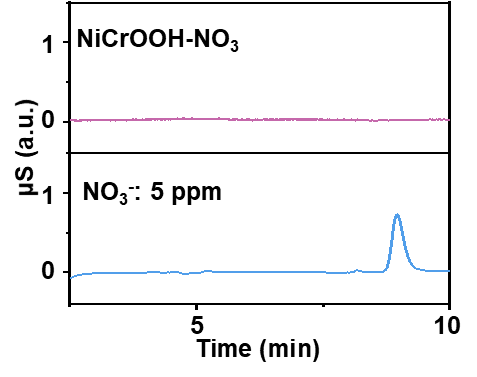


**Fig.** **S22** Ion chromatogram spectra of post-reaction supernatant and 5 ppm NO_3_^-^ solution

**
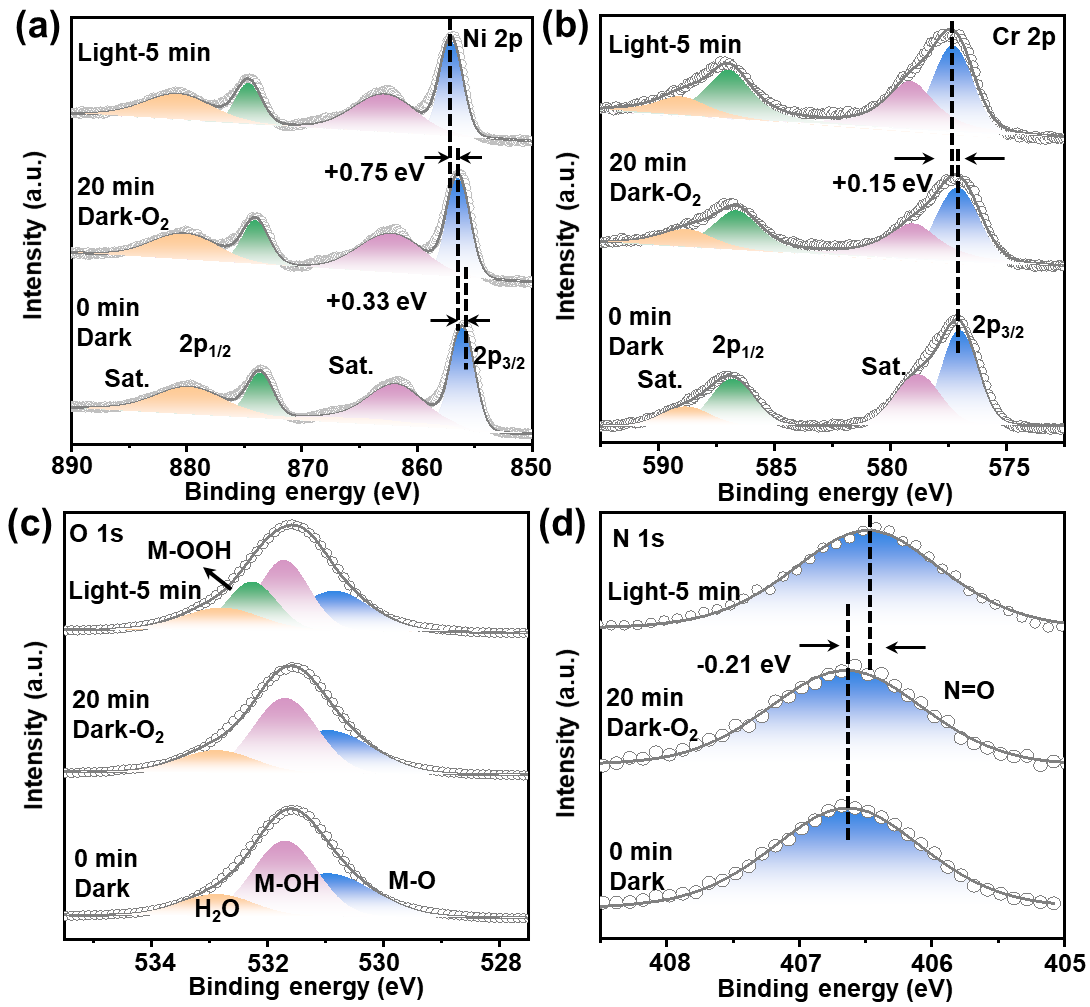
**

**Fig. S23** ISIXPS spectra of (**a**) Ni 2p, (**b**) Cr 2p, (**c**) O 1s and (**d**) N 1s of NiCrOOH-NO_3_


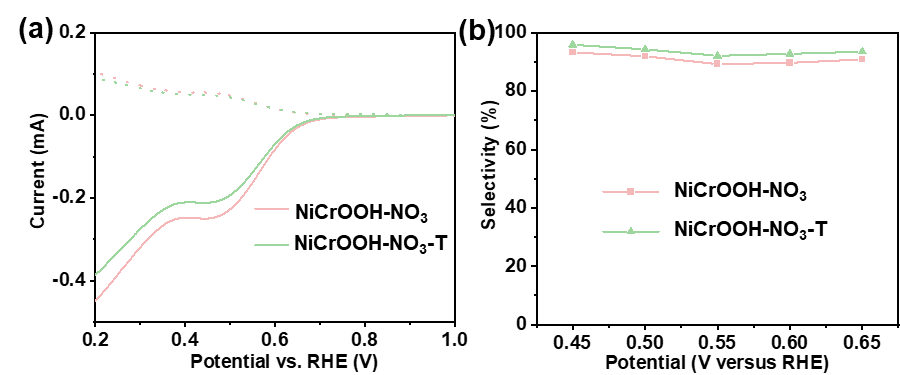


**Fig. S24** (**a**) LSV polarization curves and (**b**) H_2_O_2_ selectivities of NiCrOOH-NO_3_ and NiCrOOH-NO_3_-T


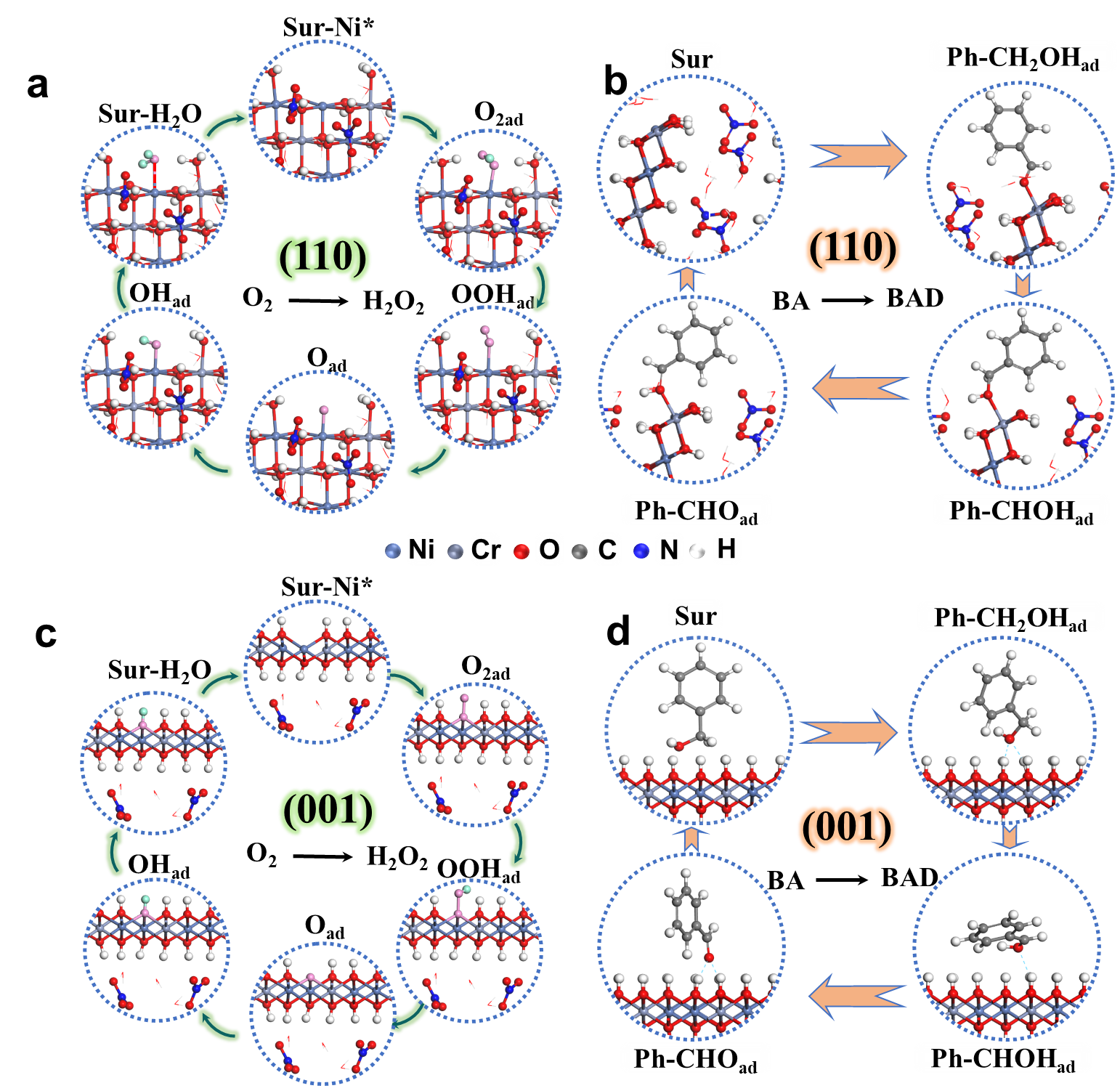


**Fig. S25** Optimized structures of the reaction intermediates on (**a, b**) (110) and (**c, d**) (001) planes over NiCrOOH-NO_3_

**
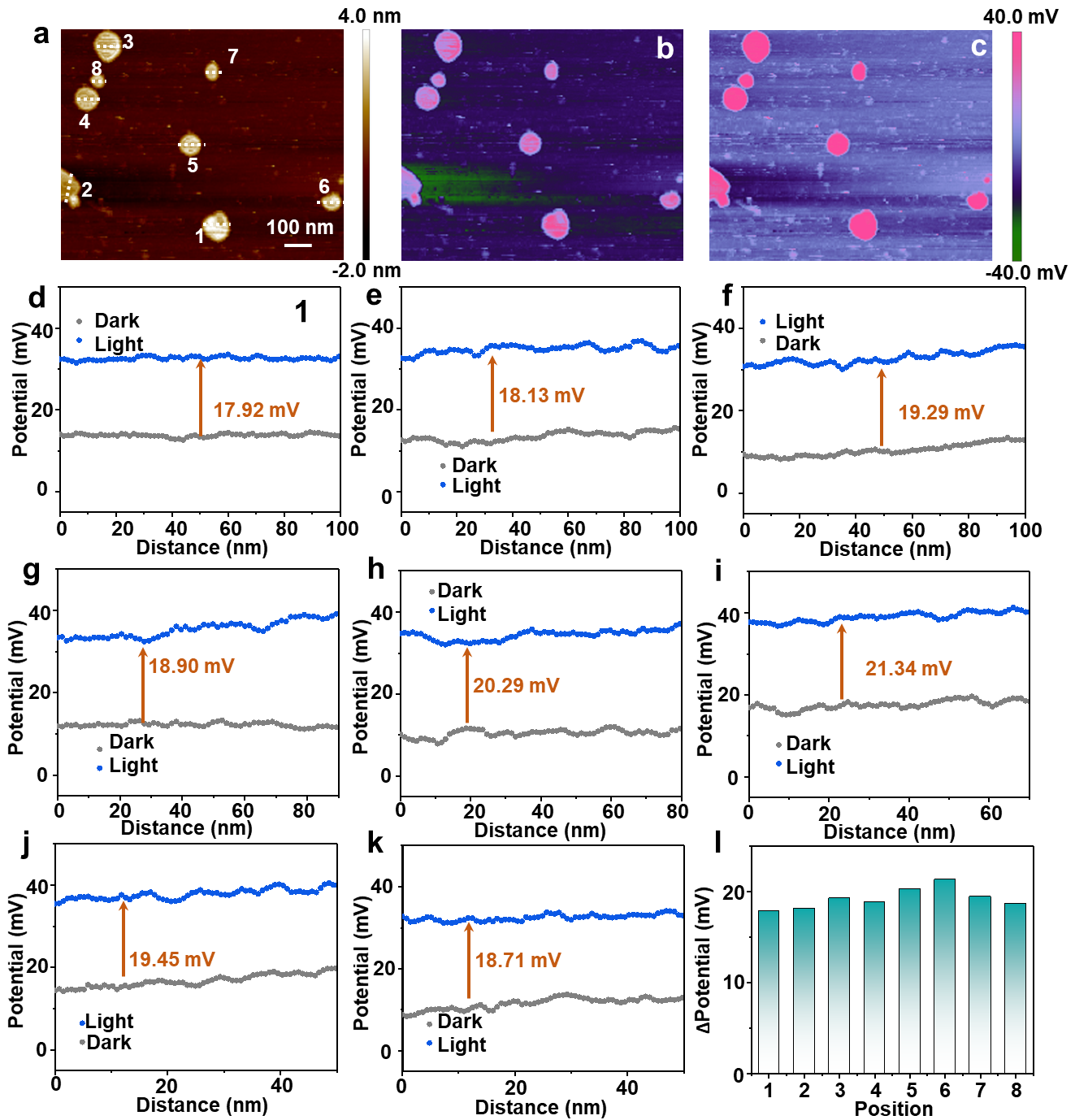
**

**Fig. S26** (**a**) Atomic force microscopy image of NiCrOOH-NO₃, corresponding KPFM images (**b**) in darkness and (**c**) under illumination, (**d-k**) line scanning surface potential profiles along the white dashed lines, (**i**) statistical potential difference of NiCrOOH-NO₃ under illumination versus dark conditions


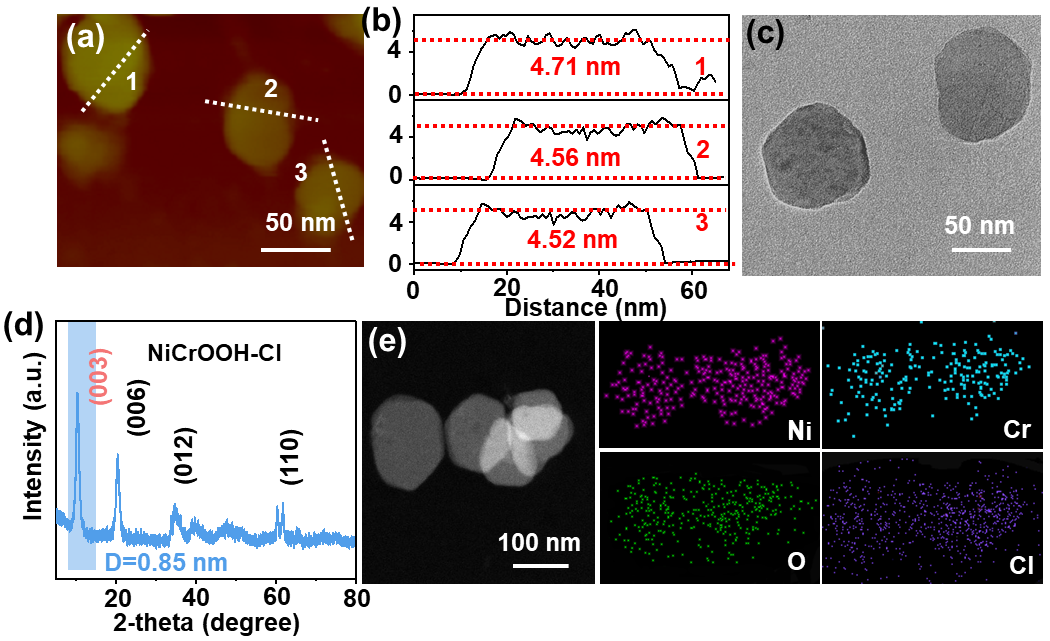


**Fig. S27** (**a**) AFM image and (**b**) height profiles of NiCrOOH-Cl nanosheets, (**c**) TEM, (**d**) XRD pattern, (**e**) High-angle annular dark-field scanning TEM image and EDS element maps for Ni, Cr, O and Cl of NiCrOOH-NO_3_-Cl

The TEM image shows that NiCrOOH-Cl has also a hexagonal plate morphology with a thickness of ~ 4.6 nm (Fig. S12a-c), close to that of NiCrOOH-NO_3_. The (003) crystal plane diffraction peak for NiCrOOH-Cl is observed at 10.28°, which corresponds to a d_003_-spacing of 0.87 nm (Fig. S12d). The HAADF and EDX elemental mapping images present the uniform distribution of Ni, Cr, Cl and O elements (Fig. S12e).


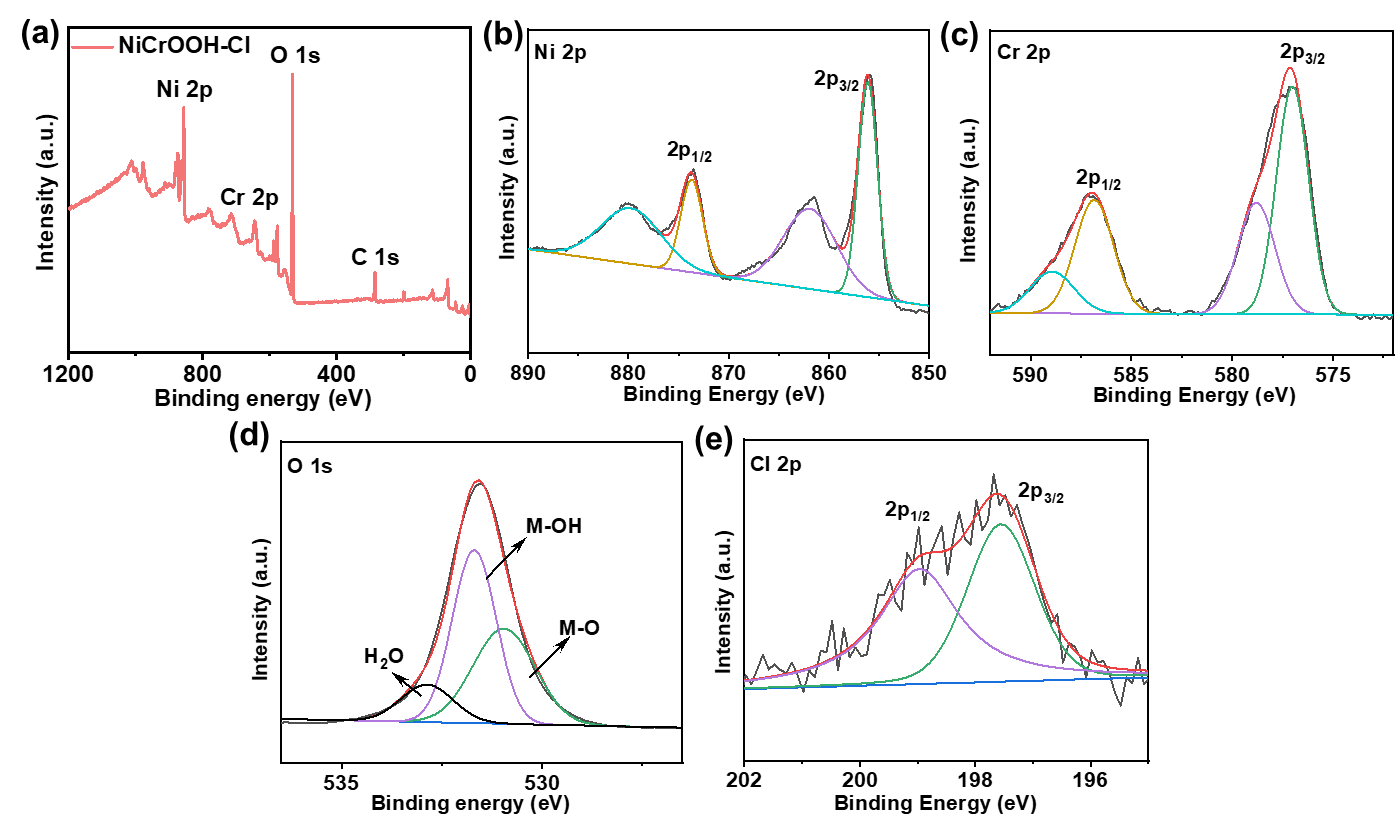


**Fig. S28** (**a**) XPS survey spectra of NiCrOOH-Cl, XPS spectra of (**b**) Ni 2p, (**c**) Cr 2p, (**d**) O 1s and (**e**) Cl 2p of NiCrOOH-Cl

The XPS survey spectra (Fig. S13a) also demonstrate the coexistence of Ni, Cr, Cl and O elements. The Ni 2p and Cr 2p peaks indicate the existence of Ni^2+^ and Cr^3+^ in NiCrOOH-Cl. Fig. S13d displays the O 2p spectrum, where two peaks are close to NiCrOOH-NO_3_. In addition, two peaks at 197.55 eV and 198.98 eV are found in the Cl 2p XPS spectra (Fig. S13e), suggesting the successful intercalation of Cl^-^ in NiCrOOH-Cl.


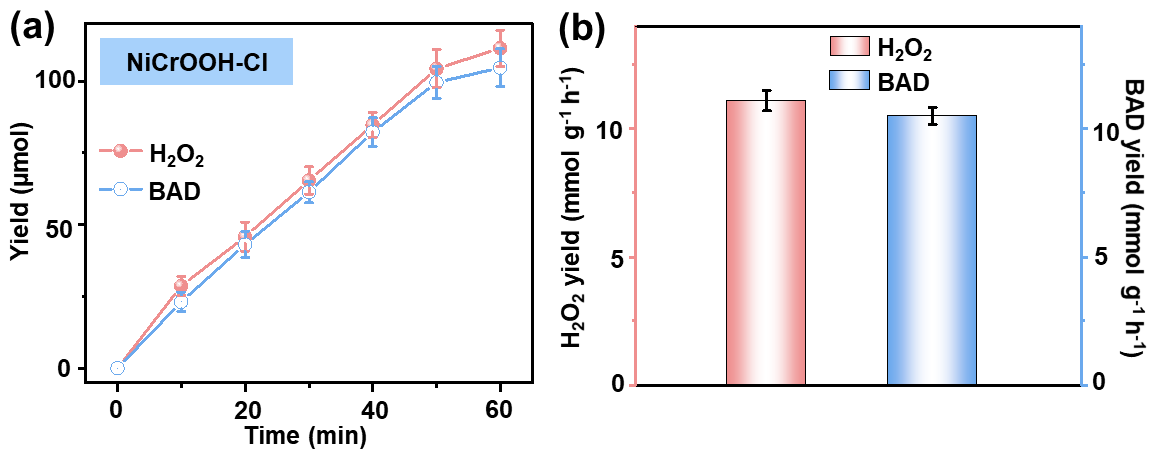


**Fig. S29** (**a**) Time course of H_2_O_2_ and BAD contents during the photocatalytic reaction over NiCrOOH-Cl, (**b**) H_2_O_2_ and BAD yield rates of NiCrOOH-Cl

**
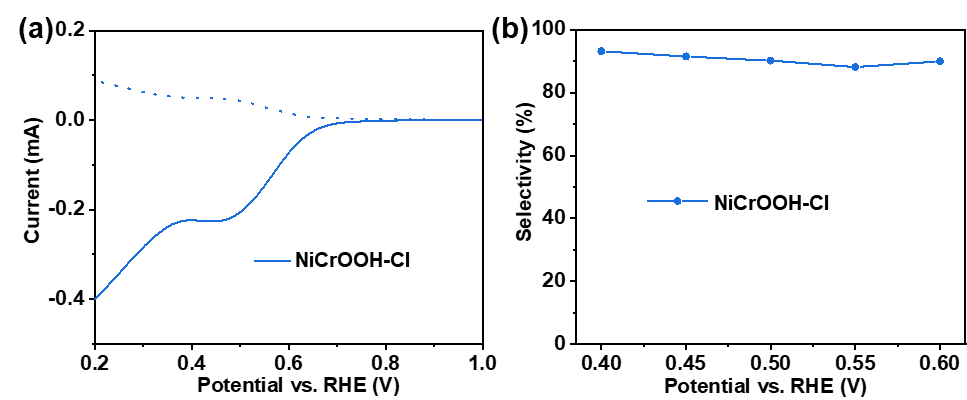
**

**Fig. S30** (**a**) LSV polarization curve and (**b**) H_2_O_2_ selectivity of NiCrOOH-Cl


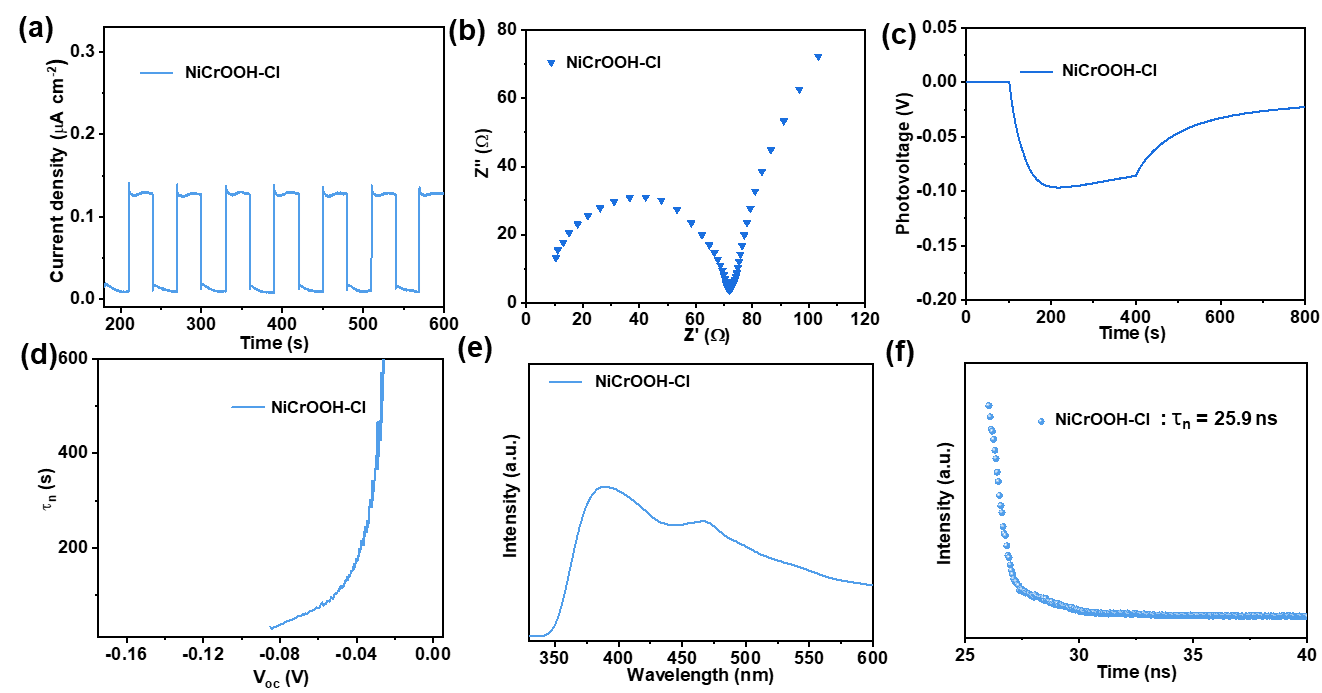


**Fig. S31** (a) transient photocurrent response, (b) EIS curve, (c) transient OCVD curve, (**d**) average electron lifetimes (τ_n_), (**e**) PL spectrum and (**f**) TRPL spectrum of NiCrOOH-Cl


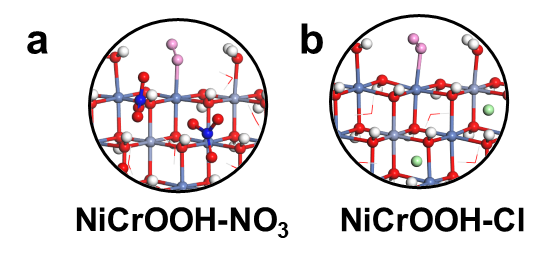


**Fig. S32** Adsorption configurations of O_2_ on NiCrOOH-NO_3_ and NiCrOOH-Cl

**Table S1** Atomic percentage of XPS for NiCrOOH-NO_3_ before and after photocatalytic reaction

| **Sample** |  | **N(%)** | **O(%)** | **Ni(%)** | **Cr(%)** | **Ni/Cr/N/O ratio from XPS** |
| --- | --- | --- | --- | --- | --- | --- |
| **Fresh** |  | 4.90%±0.98 | 56.55%  ±1.23 | 9.85%  ±1.08 | 4.89%  ±1.19 | Ni_2.00_Cr_0.99_N_0.99_O_11.48_ |
| **Used** |  | 4.86%  ±1.30 | 56.28%  ±1.27 | 9.81%  ±1.08 | 4.87%  ±1.26 | Ni_2.00_Cr_0.99_N_0.98_O_11.47_ |

**Table S2** EA and ICP-AES results of NiCrOOH-NO_3_

| **Sample** | **H(%)** | **N(%)** | **O(%)** | **Ni(%)** | **Cr(%)** | **Ni/Cr/H/N/O ratio from EA and ICP-AES** |
| --- | --- | --- | --- | --- | --- | --- |
| NiCrOOH-NO_3_ | 3.04%  ±1.09 | 3.68% ±1.37 | 48.60%  ±1.53 | 31.08%  ±1.21 | 13.60%  ±1.33 | Ni_2.00_Cr_0.99_N_0.98_O_11.47_H_11.48_ |

**Table S3** BET specific surface areas (S) of NiCrOOH-NO_3_-T, NiCrOOH-NO_3_ and NiCrOOH-Cl

| **Samples** | **S (m^2^·g^-1^)** |
| --- | --- |
| NiCrOOH-NO_3_-T | 60.2 |
| NiCrOOH-NO_3_ | 93.1 |
| NiCrOOH-Cl | 92.3 |

**Table S4** Calculated AQY and APCE value of NiCrOOH-NO_3_ under different-wavelength irradiation

| **Wavelength (nm)** | **Light power (mW)** | **Photon flux (µmol m^-2^ s⁻¹)** | **H₂O₂ production (µmol)** | **Absorptance (A)** | **AQY**  **(%)** | **APCE (%)** |
| --- | --- | --- | --- | --- | --- | --- |
| 350 | 13 | 126.24 | 22 | 0.53 | 30.84 | 58.19 |
| 400 | 15 | 159.63 | 30.4 | 0.49 | 33.70 | 68.78 |
| 450 | 18 | 215.49 | 26.07 | 0.43 | 21.41 | 49.79 |
| 500 | 20 | 266.04 | 25.43 | 0.33 | 16.92 | 51.27 |
| 550 | 23 | 336.54 | 20.93 | 0.44 | 11.00 | 25.00 |
| 600 | 16 | 255.40 | 10.58 | 0.36 | 7.33 | 20.36 |
| 650 | 13 | 224.80 | 5.87 | 0.31 | 4.62 | 14.90 |

**Table S5** Comparison H_2_O_2_ production rate of NiCrOOH-NO_3_ with recently reported photocatalysts

| **Photocatalyst** | **Light source** | **Reaction conditions**^a^ | | **Activity**  **(mmol g_cat_^-1^ h^-1^)** | | **AQY** | | **Refs.** |
| --- | --- | --- | --- | --- | --- | --- | --- | --- |
| **NiCrOOH-NO_3_** | 300 W Xe lamp  320 < λ < 780 nm | H_2_O/BA | | 28.7 | | 33.7 | | **This work** |
| QP-HPTP-COF | 300 W Xe lamp  λ > 420 nm | H_2_O | | 4.4 | | - | [1] | |
| RS | 300 W Xe lamp  λ > 420 nm | H_2_O | | 1.5 | | 13.1 | [10] | |
| rGO@MRF | 300 W Xe lamp  λ ≥ 420 nm | H_2_O | | 0.9 | | - | [43] | |
| TAPT–FTPB COF | 300 W Xe lamp  AM1.5G | H_2_O | | 3.8 | | - | [40] | |
| RF-DHAQ | 300 W Xe lamp  λ > 420 nm | H_2_O | | 1.8 | | - | [48] | |
| SA-TCPP | 300 W Xe lamp  λ > 420 nm | H_2_O | | 1.8 | | 14.9 | [47] | |
| Mo:BiVO_4_ | 300 W Xe lamp  AM 1.5G | H_2_O | | 0.1 | | 1.2 | [39] | |
| APFac | 300 W Xe lamp  λ > 420 nm | H_2_O | | 4.5 | | - | [45] | |
| COF-TfpBpy | 300 W Xe lamp | H_2_O | | 0.7 | | 13.6 | [37] | |
| PI-BD-TPB | 300 W Xe lamp | H_2_O | | 3.8 | | 14.3 | [35] | |
| CoO_x_-BCN-FeOOH | 300 W Xe lamp  λ > 420 nm | H_2_O | | 0.3 | | 8.4 | [38] | |
| Co-N@G | AM 1.5G | H_2_O | | 16.6 | | 9.1 | [44] | |
| Kf-AQ | λ > 400 nm | H_2_O | | 4.8 | | 15.8 | [46] | |
| Sb-SAPC | 300 W Xe lamp  λ > 420 nm | H_2_O | | 0.1 | | 17.6 | [42] | |
| SA-NDI | 300 W Xe lamp  AM1.5G | H_2_O/MeOH | | 1.2 | | 17.2 | [36] | |
| RF523 | λ > 420 nm | H_2_O/BA | | 0.1 | | 7.8 | [41] | |
| Pd/BMO-SOVs | blue LED | H_2_O/BA | | 0.01 | | - | [56] | |
| sonoCOF-F2 | 300 W Xe lamp  AM1.0G | H_2_O/BA | | 0.7 | | 4.8 | [57] | |
| MIL-125-NH_2_-R7 | 300 W Xe lamp  λ > 420 nm | H_2_O/BA | | 0.3 | | - | [53] | |
| Bi_2_MoO_6_-H_2_ | Xe lamp | H_2_O/BA | | 0.1 | | - | [49] | |
| OPA/Zr_92.5_Ti_7.5_-MOF | 300 W Xe lamp  λ > 420 nm | H_2_O/BA | | 13.6 | | - | [50] | |
| OPA/Fe-Zr-MOF | 500 W Xe lamp  λ > 420 nm | H_2_O/BA | | 18.3 | |  | [51] | |
| TpAzo-CPd | 300 W Xe lamp  λ > 420 nm | H_2_O/BA | | 0.7 | | - | [55] | |
| PMCR-1 | 300 W Xe lamp  λ > 420 nm | | H_2_O/BA | 5.5 | | 14 | [42] | |
| Al−TCPP | 300 W Xe lamp  AM 1.5G | | H_2_O/BA | 6.4 | | 12.1 | [58] | |
| EBA-COF | 50 W LED lamp λ > 420 nm | | H_2_O/ETOH | | 1.8 | - | [59] | |
| Tp-BTz COF | 300 W Xe lamp | | H_2_O/BA | | 100.9 | 18.0 | [60] | |
| TpPm | 300W Xe lamp  λ > 420 nm | | H_2_O/BA | | 17.0 | 22.7 | [61] | |

**Table S6** Summary of reported SCC efficiencies for photocatalytic H_2_O_2_ production

| **Samples** | **Mass(mg)** | **Volume (mL)** | | | **Temperature (°C)** | **Irradiance (mW cm^-2^)** | **Area (cm^2^)** | **Total input energy (W)** | | | **H_2_O_2_ formed (µmol)** | | **SCC** | | **Refs** |
| --- | --- | --- | --- | --- | --- | --- | --- | --- | --- | --- | --- | --- | --- | --- | --- |
| NiCrOOH-NO_3_ | 100 | | 100 | | 50 | 100 | 3.14 | 0.314 | 378.84 | | | | 3.92 | **This work** | |
| QP-HPTP-COF | 800 | | 135 | | - | 100 | 4 | 0.4 | 173.54 | | | | 1.41 | [1] | |
| RS | 500 | | 100 | | 50 | 100 | 4 | 0.400 | 172.30 | | | | 1.40 | [10] | |
| rGO@  MRF | 400 | | 150 | | 50 | 100 | 6.25 | 0.625 | 236.50 | | | | 1.23 | [43] | |
| TAPT–FTPB COF | - | | - | - | | 100 | 1 | 0.100 | 37.54 | | | | 1.22 | [40] | |
| RF-DHAQ | 400 | | 150 | 50 | | 100 | 4 | 0.400 | 147.69 | | | | 1.20 | [48] | |
| SA-TCPP | - | | - | 80 | | 100 | 1.09 | 0.109 | | 36.89 | | 1.1 | | [47] | |
| SA-NDI | 150 | | 40 | 60 | | 100 | 3.14 | 0.314 | 99.51 | | | | 1.03 | [36] | |
| COF-TfpBpy | 600 | | 400 | 60 | | 40.8 | 16 | 0.653 | 531.70 | | | | 1.08 | [37] | |
| PI-BD-TPB | 80 | | 60 | 50 | | 100 | 0.79 | 0.0785 | 74.07 | | | | 0.92 | [35] | |
| CoO_x_-BCN-FeOOH | - | | - | - | | 100 | 4 | 0.4 | 92.31 | | | | 0.75 | [38] | |
| Co-N@G | 200 | | - | - | | 100 | - | - | 707.80 | | | | 0.72 | [44] | |
| Kf-AQ | 5 | | 30 | -- | | 98.4 | 0.37 | 0.037 | 7.97 | | | | 0.7 | [46] | |
| Sb-SAPC | 500 | | 100 | - | | 100 | 1 | 0.1 | | 18.77 | | 0.61 | | [42] | |
| APFac | 400 | | 150 | 50 | | 100 | 4 | 0.400 | | 66.46 | | 0.54 | | [45] | |
| RF523 | 250 | | 50 | 60 | | 100 | 3.14 | 0.314 | | 48.31 | | 0.5 | | [41] | |
| Mo:  BiVO_4_ | - | | 12 | - | | 100 | 1.91 | 0.191 | | 17.04 | | 0.29 | | [39] | |
| Tp-BTz COF | 5 | | 20 | 25 | | 100 | 12.6 | 1.26 | | 569.91 | | 1.47 | | [60] | |
| TpPm | 150 | | 50 | - | | 100 | 12.56 | 1.26 | | 356.68 | | 1.84 | | [61] | |

**Table S7** ICP-AES results of the reaction solution after photocatalytic test

| **Sample** | **Ni (mg/L)** | **Cr (mg/L)** |
| --- | --- | --- |
| Reaction solution  after test | 0.010 | 0.012 |

**Supplementary References**

1. T. Takata, J. Jiang, Y. Sakata, M. Nakabayashi, N. Shibata et al., Photocatalytic water splitting with a quantum efficiency of almost unity. Nature **581**(7809), 411–414 (2020). <https://doi.org/10.1038/s41586-020-2278-9>
2. J. Meng, Y. Duan, S. Jing, J. Ma, K. Wang et al., Facet junction of BiOBr nanosheets boosting spatial charge separation for CO_2_ photoreduction. Nano Energy **92**, 106671 (2022). <https://doi.org/10.1016/j.nanoen.2021.106671>
3. Y. Zhang, Y. Li, X. Xin, Y. Wang, P. Guo et al., Internal quantum efficiency higher than 100% achieved by combining doping and quantum effects for photocatalytic overall water splitting. Nat. Energy **8**(5), 504–514 (2023). <https://doi.org/10.1038/s41560-023-01242-7>
4. S.-D. Huang, C. Shang, P.-L. Kang, X.-J. Zhang, Z.-P. Liu, LASP: Fast global potential energy surface exploration. Wires Comput. Mol. Sci. **9**(6), e1415 (2019). <https://doi.org/10.1002/wcms.1415>
5. G. Kresse, J. Furthmüller, Efficiency of ab-initio total energy calculations for metals and semiconductors using a plane-wave basis set. Comput. Mater. Sci. **6**(1), 15–50 (1996). <https://doi.org/10.1016/0927-0256(96)00008-0>
6. P.E. Blöchl, Projector augmented-wave method. Phys. Rev. B **50**(24), 17953–17979 (1994). <https://doi.org/10.1103/physrevb.50.17953>
7. G. Kresse, D. Joubert, From ultrasoft pseudopotentials to the projector augmented-wave method. Phys. Rev. B **59**(3), 1758–1775 (1999). <https://doi.org/10.1103/physrevb.59.1758>
8. J.P. Perdew, K. Burke, M. Ernzerhof, Generalized gradient approximation made simple. Phys. Rev. Lett. **77**(18), 3865–3868 (1996). <https://doi.org/10.1103/physrevlett.77.3865>
9. S.L. Dudarev, G.A. Botton, S.Y. Savrasov, C.J. Humphreys, A.P. Sutton, Electron-energy-loss spectra and the structural stability of nickel oxide:   An LSDA+U study. Phys. Rev. B **57**(3), 1505–1509 (1998). <https://doi.org/10.1103/physrevb.57.1505>
10. N.J. Mosey, P. Liao, E.A. Carter, Rotationally invariant *ab initio* evaluation of Coulomb and exchange parameters for DFT+U calculations. J. Chem. Phys. **129**, 014103 (2008). <https://doi.org/10.1063/1.2943142>
11. P. Liao, J.A. Keith, E.A. Carter, Water oxidation on pure and doped hematite (0001) surfaces: prediction of co and Ni as effective dopants for electrocatalysis. J. Am. Chem. Soc. **134**(32), 13296–13309 (2012). <https://doi.org/10.1021/ja301567f>
12. S. Grimme, J. Antony, S. Ehrlich, H. Krieg, A consistent and accurate *ab initio* parametrization of density functional dispersion correction (DFT-D) for the 94 elements H-Pu. J. Chem. Phys. **132**(15), 154104 (2010). <https://doi.org/10.1063/1.3382344>
13. S. Grimme, S. Ehrlich, L. Goerigk, Effect of the damping function in dispersion corrected density functional theory. J. Comput. Chem. **32**(7), 1456–1465 (2011). <https://doi.org/10.1002/jcc.21759>
14. H.J. Monkhorst, J.D. Pack, Special points for Brillouin-zone integrations. Phys. Rev. B **13**(12), 5188–5192 (1976). <https://doi.org/10.1103/physrevb.13.5188>
15. K. Mathew, R. Sundararaman, K. Letchworth-Weaver, T.A. Arias, R.G. Hennig, Implicit solvation model for density-functional study of nanocrystal surfaces and reaction pathways. J. Chem. Phys. **140**(8), 084106 (2014). <https://doi.org/10.1063/1.4865107>
16. K. Mathew, V.S. Chaitanya Kolluru, S. Mula, S.N. Steinmann, R.G. Hennig, Implicit self-consistent electrolyte model in plane-wave density-functional theory. J. Chem. Phys. **151**(23), 234101 (2019). <https://doi.org/10.1063/1.5132354>
17. G.-F. Wei, C. Shang, Z.-P. Liu, Confined platinum nanoparticle in carbon nanotube: structure and oxidation. Phys. Chem. Chem. Phys. **17**(3), 2078–2087 (2015). <https://doi.org/10.1039/c4cp04145c>
18. 18 CRC Handbook of Chemistry and Physics; 84th ed.; Lide, D. R., Ed.; CRC Press: Boca Raton, FL, 2003−2004.
